# A multifunctional near‐infrared dual‐channel off‐on‐off probe for sequential detection of fluoride and hypochlorous acid: Multimatrix applications

**DOI:** 10.1002/smo2.70092

**Published:** 2026-08-17

**Authors:** Tongtong Li, Xiaodong Zhang, Tong Yu, Dong Cai, Yabai Li, Shiyu Wu, Jingyi Gao, Yaqian Xu, Yihui Sun, Xuewei Pu, Fanjie Meng

**Affiliations:** ^1^ College of Pharmacy Jinzhou Medical University Jinzhou China; ^2^ Linyi Vocational University of Science and Technology Linyi China; ^3^ School of Chemical Engineering Shandong Institute of Petroleum and Chemical Technology Dongying China

**Keywords:** environmental monitoring, fluoride ion and hypochlorous acid probe, multi‐response, NIR probe, sequential recognition

## Abstract

Fluoride (F^−^) and hypochlorous acid (HClO) are chemically distinct yet environmentally and biologically critical analytes. F^−^ is widespread in soil, water, and food additives, whereas HClO acts as a key in vivo immune molecule and food disinfectant; abnormal levels of either pose significant risks. Herein, we report a novel multifunctional near‐infrared (NIR) dual‐channel probe (DHM) for sequential F^−^/HClO detection with an “off‐on‐off” response. The tert‐butyldiphenylsilyl (TBDPS) moiety in DHM quenches its initial fluorescence (“off” state). F^−^ specifically cleaves TBDPS, generating a phenolate group with enhanced electron‐donating capacity that activates intramolecular charge transfer, switching DHM to “on” with dramatically enhanced NIR red fluorescence. Subsequently, HClO oxidizes the malononitrile moiety of the F^−^‐activated product, disrupting conjugation and inducing a red‐to‐blue switch with a massive 190 nm spectral separation, thereby achieving zero signal crosstalk. DHM exhibits rapid response, excellent anti‐interference ability, and robust performance in complex matrices. Crucially, this strict “first F^−^, then HClO” dependency perfectly aligns with the pathological timeline of fluoride‐induced oxidative stress and the practical demands of municipal water monitoring. It has been successfully applied for dual‐channel imaging in Huh7 cells and mice, as well as quantitative detection in water/food samples with satisfactory results, showing great potential in related fields.

## Graphical abstract

This multifunctional, sequence‐specific probe (DHM) enables stepwise detection: fluoride triggers red/near‐infrared emission via cleavage, then hypochlorous acid induces blue fluorescence. It achieves fast, selective visual detection in environmental samples, foods, and in vivo models.

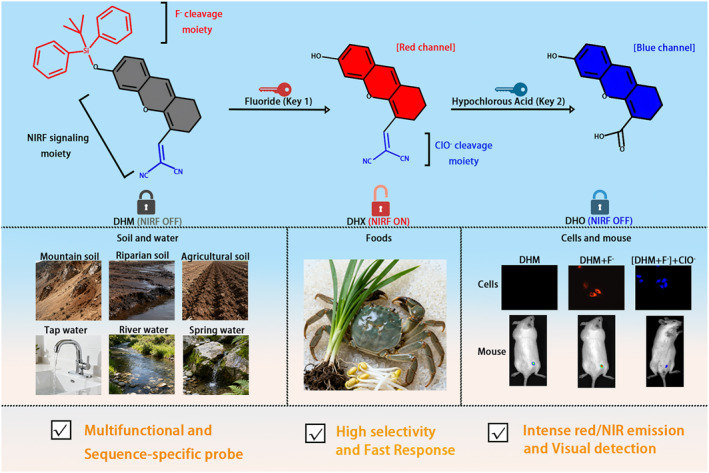

## 1 INTRODUCTION

Fluoride (F^−^) and hypochlorous acid (HClO) are critical analytes that play pivotal roles in biology, environmental science, and the food industry[^1^, ^2^, ^3^, ^4^]: physiologically, F^−^ facilitates bone mineralization and maintains dental health, whereas HClO acts as a key antimicrobial agent in the innate immune system[^5^, ^6^]; from an environmental perspective, F^−^ is ubiquitously present in soil and water, and HClO is widely employed for water disinfection, though excessive levels of both can induce substantial environmental hazards[^7^, ^8^]; and in the food industry, F^−^ is occasionally incorporated into food additives while HClO serves as a prevalent sanitizer.[^9^, ^10^] The World Health Organization and other regulatory authorities have thus established stringent exposure limits for these analytes, as overexposure to F^−^ is associated with fluorosis and other adverse health outcomes, while excessive HClO can cause cellular/tissue damage and contribute to the development of inflammatory and degenerative diseases[^11^, ^12^, ^13^, ^14^, ^15^]; notably, the pathogenesis of fluorosis involves reactive oxygen species (ROS)‐mediated oxidative stress, and hypochlorite (HClO)‐he conjugate base of HClO‐is recognized as a critical ROS, and as a potent oxidant, HClO can oxidize cellular biomolecules and catalyze the generation of free radicals,[^16^, ^17^, ^18^] thereby establishing a link between F^−^ overexposure and HClO‐related oxidative damage. Therefore, the development of robust analytical methods for the precise quantification of F^−^ and HClO in various matrices is of great scientific significance and practical value.

In recent years, fluorescent probes have become an increasingly prominent research tool in analytical and biomedical fields, driven by their unique advantages: high spatial resolution for pinpointing target analytes, outstanding sensitivity for trace detection, and noninvasive characteristics that enable real‐time monitoring in living systems—features that traditional analytical methods (e.g., chromatography, ion‐selective electrodes) often lack.[^19^, ^20^, ^21^, ^22^] To date, a range of fluorescent probes has been reported for the detection of F^−^ and HClO individually, with applications covering biological samples (e.g., cells, tissues), environmental water matrices, and food products.[^23^, ^24^, ^25^, ^26^] However, three critical limitations continue to hinder the practical advancement of this technology. First, the acquisition of probes with optimal optical properties‐such as long wavelength emission (which reduces autofluorescence interference in complex samples)‐frequently relies on complex synthetic routes and labor intensive purification processes, increasing production costs and limiting scalability.[^27^, ^28^, ^29^, ^30^] Second, the development of dual‐response fluorescent probes capable of sequential and distinguishable detection of F^−^ and HClO remains a major challenge; most existing dual‐analyte probes either lack clear spectral separation between the two targets or suffer from cross interference, compromising detection accuracy.[^31^, ^32^, ^33^] To fundamentally solve this, there is a compelling need for cascade‐type probes with structurally dependent sequential responses—where the structural transformation induced by the first analyte generates a mandatory intermediate for the second. This “locked” sequential mechanism essentially eliminates signal crosstalk and unlocks the potential for ultra‐large spectral separation. Third, very few probes exhibit the versatility to perform reliable detection of F^−^ and HClO across diverse matrices (live organisms, water, and food).[^34^, ^35^, ^36^] In real world samples, complex components (e.g., competing ions in soil/water, biomacromolecules in cells, or organic additives in food) often disrupt probe sensitivity and selectivity, rendering most probes suitable for only a single type of matrix.[^37^, ^38^, ^39^] Thus, the design and synthesis of a dual‐response fluorescent probe that can sequentially monitor F^−^ and HClO with high performance across biological, environmental, and food matrices are urgently needed to address these unmet demands.

To tackle these unresolved challenges, we herein describe the design and synthesis of a novel multi‐response fluorescent probe (DHM) tailored for the sequential and discriminative detection of F^−^ and HClO via a deliberate cascade dual‐response mechanism. DHM is constructed around a dihydroxanthene core, incorporating a silicon‐containing functional group as the specific recognition site for F^−^. Crucially, its redox‐active malononitrile moiety remains electronically “locked” and inert until F^−^ triggers the cleavage of the silyl group, thereby generating an activated intermediate strictly susceptible to subsequent HClO‐induced oxidation. Notably, DHM offers a streamlined synthetic pathway with high yields, paired with exceptional sensing capabilities: a massive 190 nm spectral separation that guarantees zero signal crosstalk, rapid response dynamics (20 s for F^−^, 3 min for HClO), ultra‐low limits of detection (LOD) (14 nM for F^−^, 3.19 μM for HClO), and robust selectivity against a wide range of common interfering species (including an intrinsic non‐response to HClO in the absence of F^−^, thereby ensuring strict sequential fidelity) in complex matrices. Beyond in vitro characterization, DHM has been successfully validated for tracking the F^−^‐triggered HClO cascade in Huh7 cells through dual channels (red for F^−^, blue for HClO) and for in vivo fluorescence imaging in mouse models. Furthermore, the probe exhibits reliable quantitative performance for F^−^ and HClO detection across diverse real‐world samples, including environmental matrices (soil, tap water, river water, spring water), food products (Chinese mitten crab, soybean sprouts, *Houttuynia cordata*, spring onion, rice), and portable sensing applications via DHM‐loaded cotton swabs. This structurally locked cascade mechanism enables the strict F^−^‐gated sequential and differentiated detection of F^−^ and HClO in natural matrices and biological environments, thus providing a novel, high‐fidelity tool for the sequential tracking of these two interconnected target analytes.

## 2 EXPERIMENTAL SECTION

All materials, instruments, cell culture, imaging experiments, and water treatment procedures, along with the experimental protocol for the smartphone application and the analysis of food and daily necessities, are available in Supporting Information S1.

## 3 RESULTS AND DISCUSSION

### 3.1 Molecular design of probe DHM

Details regarding the specific synthesis of the DHM fluorescent probe are provided in Scheme S1 of Supporting Information S1. The structural characterization of DHM and DHX was confirmed by ^1^H nuclear magnetic resonance (^1^H NMR) spectroscopy, and DHX was additionally characterized by ^13^C NMR spectroscopy (Figures S11–S14). The dihydroxanthene moiety of DHM acts as the fluorophore of this fluorescent probe. Its silicon‐containing group acts as a specific recognition site for F^−^, initiating a reaction that generates DHX. Subsequently, DHX undergoes selective oxidation by HClO, during which the carbon‐carbon double bond within its reactive moiety is preferentially oxidized, thereby facilitating the formation of DHO. All three species (DHM, DHX, and DHO) retain the dihydroxanthene scaffold but exhibit distinct structural and reactivity profiles, enabling the probe to sequentially respond to F^−^ and HClO via specific chemical transformations. Thus, DHM enables the sequential detection of F^−^ and HClO via the formation of distinct reaction products specific to each analyte.

### 3.2 Single crystal X‐ray analysis

**FIGURE 1:**
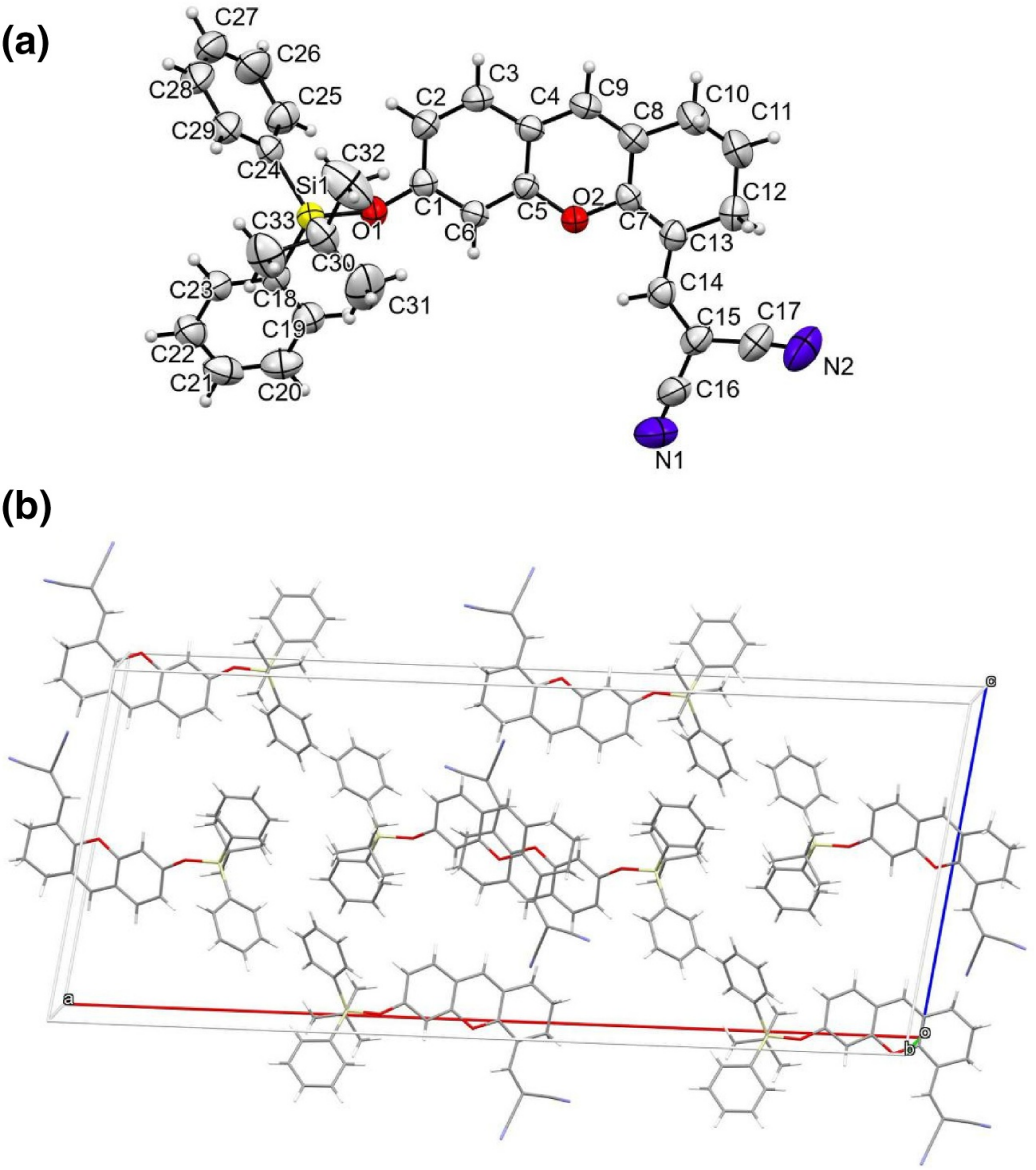
Thermal ellipsoids of DHM are drawn at 50% probability (a). Crystal packing of the structure DHM. Hydrogen atoms are omitted for clarity (b).

The structure was resolved by the SHELXTL program, and the structural information of the probe was deposited at the Cambridge Crystallographic Data Center (CCDC 2482607). X‐ray crystallographic analysis of DHM (Table 1, Figure 1) provides crucial insights into its structural features and crystallographic behavior, which are pivotal for understanding its sensing mechanism. DHM crystallizes in a monoclinic crystal system with the C2/c space group, featuring a formula weight of 514.68, an asymmetric unit with *Z* = 8, unit cell dimensions (*a* = 41.4723(13) Å, *b* = 7.8648(3) Å, *c* = 17.3452(6) Å, *β* = 98.9240(10)°), a volume of 5589.0(3) Å^3^, a calculated density of 1.223 g/cm^3^, and an absorption coefficient of 0.116 mm^−1^. Structural refinement parameters (*R*_1_ = 0.0514 for *I* ≥ 2*σ*(*I*), goodness‐of‐fit on *F* ^2^ = 1.030) confirm high data quality. The molecular structure (Figure 1a) reveals the spatial arrangement of the dihydroxanthene core, silicon‐containing aromatic moiety, and cyano groups, with the thermal ellipsoid plot (50% probability) highlighting the conformational flexibility of the silicon‐substituted group, which is crucial for F^−^ recognition. Meanwhile, the crystal packing (Figure 1b, hydrogen atoms omitted) exhibits a dense lattice dominated by van der Waals forces (lacking strong hydrogen bonding or π‐π stacking) that facilitates analyte diffusion. Further insight into the molecular conformation of DHM is provided by its selected bond lengths (Å), bond angles (°), and torsion angles (°) derived from X‐ray diffraction measurements (Table S3). These features‐including the monoclinic system, bulky substituent arrangement, and lattice packing‐rationalize DHM's selective response to F^−^ (via the silicon‐containing recognition site) and subsequent reactivity with HClO, validating its design as a sequential fluorescent probe for dual‐analyte sensing.

**TABLE 1:** X‐ray crystallographic structural refinement data of crystal for DHM.

Parameter	Compound (DHM)
Empirical formula	C_33_H_30_N_2_O_2_Si
Formula weight	514.68
Temperature/K	273.15
Crystal system	Monoclinic
Space group	C2/c
Unit cell dimensions	*a* = 41.4723(13) Å, alpha = 90°
	*b* = 7.8648(3) Å, beta = 98.9240(10)°
	*c* = 17.3452(6) Å, gamma = 90°
Volume	5589.0(3) Å^3^
*Z*	8
Calculated density	1.223 g/cm^3^
Absorption coefficient	0.116 mm^−1^
*F* (000)	2176.0
Crystal size	0.3 × 0.2 × 0.15 mm^3^
Theta range for data collection	4.754–50.686
Limiting indices	−49 ≤ *h* ≤ 49, −9 ≤ *k* ≤ 9, −20 ≤ *l* ≤ 20
Reflections collected	37,308
Independent reflections	5122 [*R*_int_ = 0.0731, *R*_sigma_ = 0.0407]
Data/restraints/parameters	5122/0/347
Goodness‐of‐fit on *F* ^2^	1.030
Final *R* indexes [*I* ≥ 2*σ*(*I*)]	*R*_1_ = 0.0514, *wR*_2_ = 0.1182
Final *R* indexes (all data)	*R*_1_ = 0.0859, *wR*_2_ = 0.1388
Largest diff. peak and hole	0.49/−0.35 eÅ^−3^

### 3.3 Optical performance of probe DHM towards F^−^ and HClO

**FIGURE 2:**
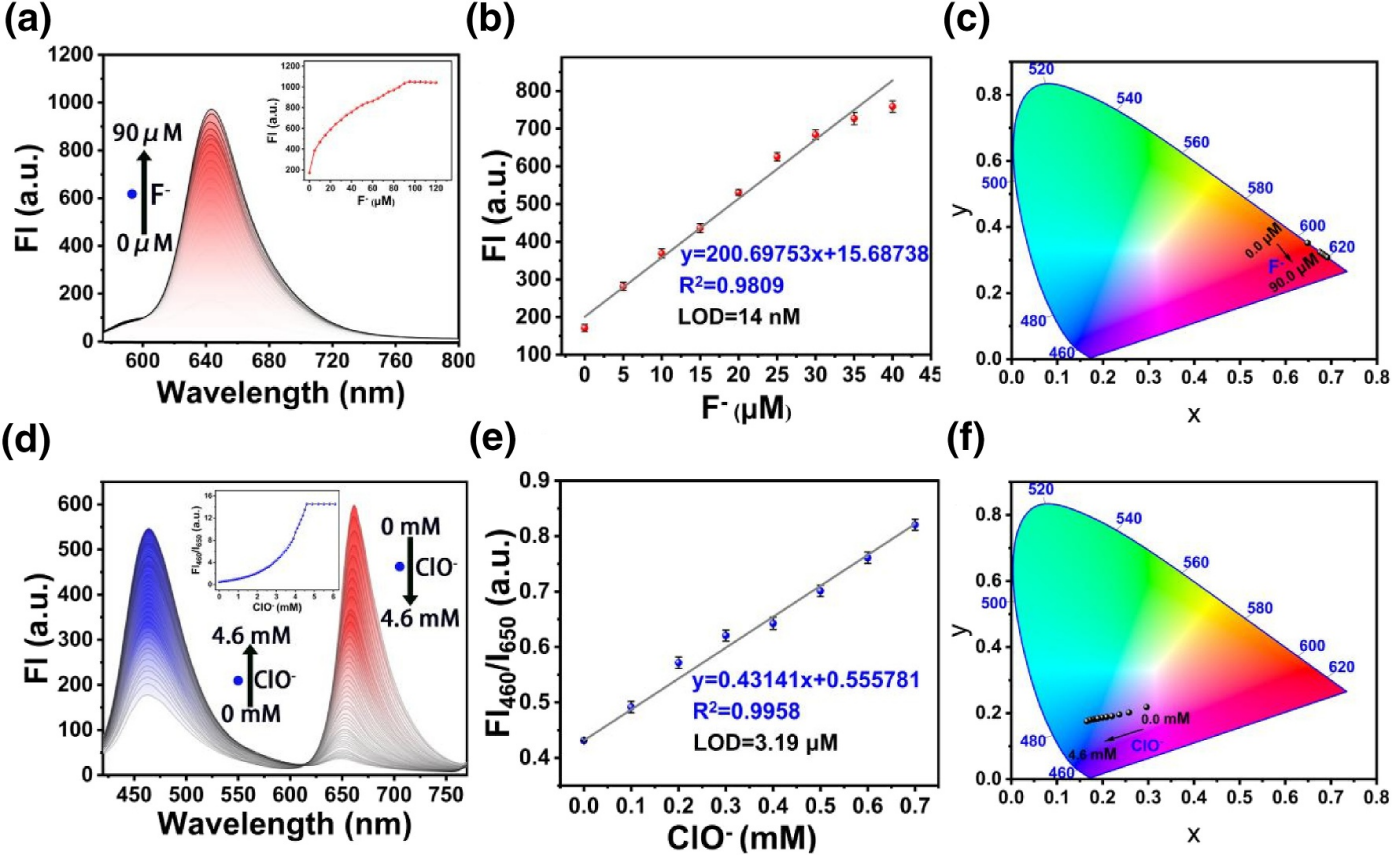
The fluorescence emission spectra of the probe DHM (5 μM) treated with different concentrations of F^−^ (0–90 μM) (a, *λ*_ex_ = 550 nm, *λ*_em_ = 650 nm), and the linear relationship between the fluorescence emission intensity and the concentration of F^−^ (b, 0–40 μM). The fluorescence in the CIE diagram of the probe changes with the increase in the added F^−^ concentration (c). The fluorescence emission spectra of DHM (5 μM) upon addition of F^−^ (120 μM), followed by treatment with HClO at different concentrations (0–4.6 mM) (d, *λ*_ex_ = 390 nm, *λ*_em_ = 460/650 nm), and the linear relationship between the fluorescence emission intensity and the concentration of HClO (e, 0–0.7 mM). The fluorescence in the CIE diagram of the probe changes with the increase in the added HClO concentration (f). Data are presented as mean ± SD (*n* = 3). HClO, hypochlorous acid.

We optimized the performance of probe DHM for F^−^ in various solvent systems, and the results indicated that it exhibited the best performance in a DMSO/H_2_O (7/3, v/v) mixture (Figures S1 and S2). Building on this optimized condition, the fluorescence sensing properties of DHM toward F^−^ and HClO were systematically investigated, and the results provide compelling evidence for its utility as a sequential dual‐analyte probe: for F^−^ detection (Figure 2), the fluorescence emission spectra show a significant enhancement at 650 nm (*λ*_ex_ = 550 nm) with increasing F^−^ concentration (0–90 μM) (Figure 2a), accompanied by a clear red‐shift in the CIE diagram (Figure 2c). This response is attributed to the specific cleavage of the silicon–oxygen bond by F^−^ that triggers the formation of a conjugated species, while the linear relationship between fluorescence intensity and F^−^ concentration (0–40 μM, Figure 2b) yields a low LOD of 14 nM‐calculated using the formula LOD = 3*σ*/*k* (*σ*: standard deviation of blank measurements; *k*: slope of the linear plot of fluorescence intensity vs. analyte concentration).^[40]^ This value is well below physiological and environmentally relevant levels of F^−^, indicating DHM's high sensitivity for F^−^ sensing.[^41^, ^42^] Subsequently, evaluation of the [DHM + F^−^] system's sensing behavior toward HClO revealed that upon the addition of HClO (0–4.6 mM) to the conjugated species formed after F^−^ interaction (Figure 2d), the fluorescence emission at 460 nm (*λ*_ex_ = 390 nm) increased prominently while that at 650 nm decreased, accompanied by a distinct color change in the CIE diagram (Figure 2f). This dual‐wavelength response originates from the oxidation of the conjugated species by HClO to form a new species with altered electronic conjugation. The linear correlation between fluorescence intensity and HClO concentration (0–0.7 mM, Figure 2e) yields a LOD of 3.19 μM, which confirms that DHM possesses satisfactory sensitivity for HClO detection following sequential recognition of F^−^.

Notably, the sequential sensing mechanism, wherein F^−^ initiates the primary reaction to generate a reaction intermediate that subsequently undergoes a response to HClO, endows DHM with the capability to discriminate between these two analytes through distinct spectral and chromatic alterations. The fluorescence intensity of DHM exhibits a rapid increase within 20 s upon the addition of F^−^, followed by a plateau (*λ*_em_ = 650 nm), thereby demonstrating rapid and stable kinetic behavior for F^−^ sensing (Figure S3); the [DHM + F^−^] system manifests an optimal fluorescence response within a neutral to weakly alkaline pH range (pH 7–10), a characteristic advantageous for potential applications in physiological or environmental matrices (Figure S4); for the detection of HClO subsequent to F^−^ recognition, the [DHM + F^−^] system responds to HClO within 3 min (*λ*_em_ = 460 nm), with the fluorescence intensity increasing and then stabilizing (Figure S5), indicative of efficient sensing kinetics for HClO. The low LODs for both analytes, in conjunction with distinct transitions in fluorescence spectra and CIE diagrams, underscore its potential for practical dual‐analyte detection scenarios, such as environmental monitoring or biological systems where F^−^ and HClO may coexist or appear sequentially, while robust linear relationships and unique spectral responses further validate its reliability and selectivity. Compared to numerous reported single and dual‐analyte probes (Tables S4–S6), DHM exhibits superior sensing performance, including lower detection limits, distinguishable spectral responses, and favorable response kinetics, thereby highlighting its merits in the sequential detection of F^−^ and HClO.

### 3.4 Selectivity and anti‐interference ability of probe DHM

**FIGURE 3:**
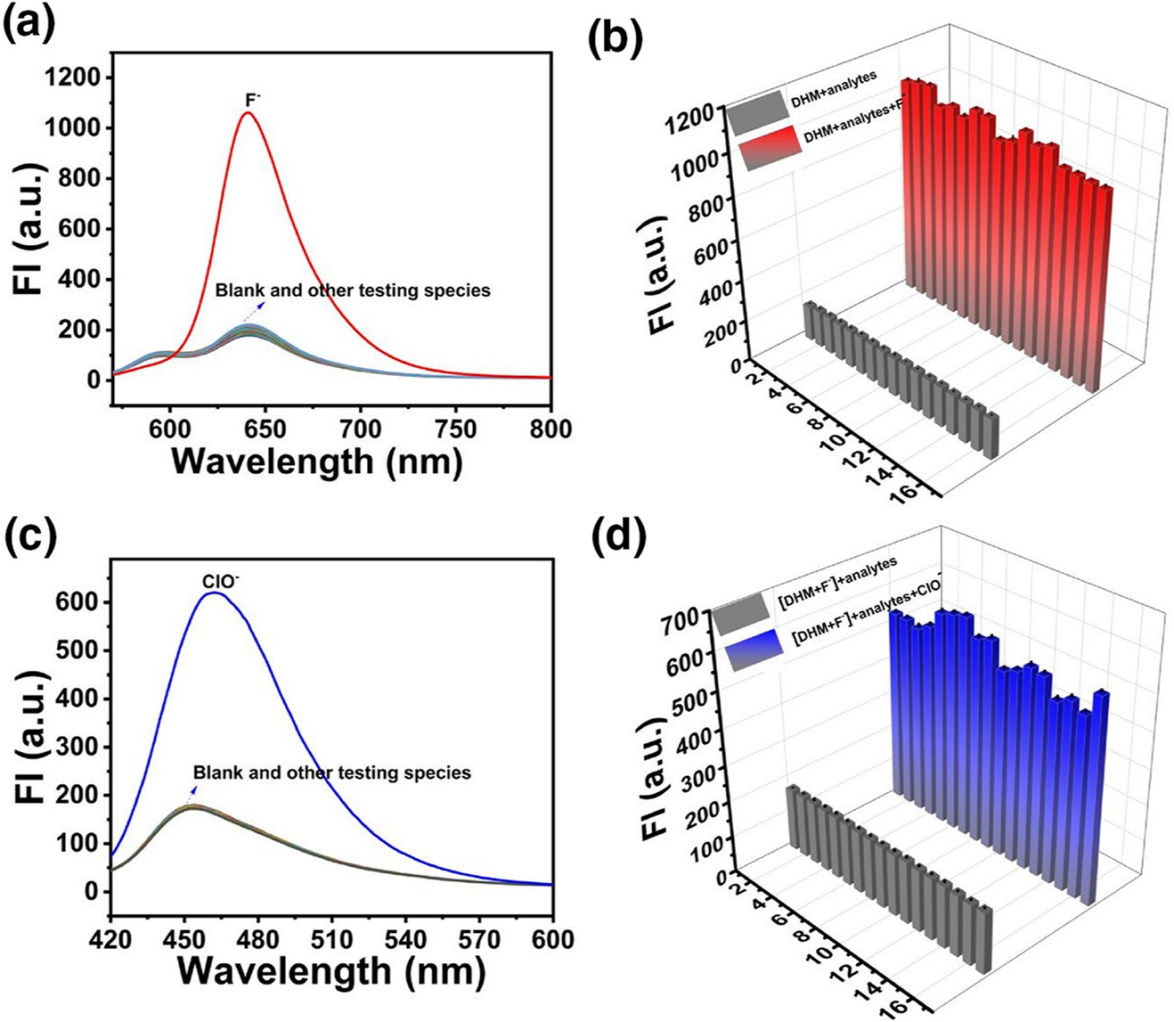
Fluorescence spectra (a, c) and fluorescent intensities (b, d) of probe DHM (5.0 μM) and DHM (5.0 μM) + F^−^ (120 μM) after the addition of various species for 40 min. *λ*_em_ = 650 nm for panels (a) and (b); *λ*_em_ = 460 nm for panels (c) and (d). (a, b): 1, Blank; 2, F^−^; 3, Br^−^; 4, Cl^−^; 5, I^−^; 6, SCN^−^; 7, NO_3_^−^; 8, ClO_4_^−^; 9, HCO_3_^−^; 10, HSO_3_^−^; 11, CO_3_^2−^; 12, SO_4_^2−^; 13, CN^−^; 14, H_2_PO_4_^2−^; 15, ACO^−^; 16, N_3_^−^; 17, NO_2_^−^. (c, d): 1, Blank; 2, HClO; 3, HCO_3_^−^; 4, SO_4_^2−^; 5, NO_3_^−^; 6, ^−^O_2_; 7, TBHP; 8, ROO.; 9, ·OH; 10, ACO^−^; 11, Br^−^; 12, F^−^; 13, Cl^−^; 14, I^−^; 15, Cys; 16, Hcy; and 17, Glutathione. Data are presented as mean ± SD (*n* = 3).

To sequentially evaluate the selectivity of probe DHM toward F^−^ and HClO, the probe response to various interfering species was investigated. As visualized in the fluorescent images (Figure S6), distinct fluorescence color changes occurred sequentially with the successive addition of the two targets, directly demonstrating DHM's sequential recognition of F^−^ and HClO. As shown in Figure 3a,b (*λ*_em_ = 650 nm), among species like Br^−^, Cl^−^, I^−^, SCN^−^, NO_3_^−^, ClO_4_^−^, HCO_3_^−^, HSO_3_^−^, CO_3_^2−^, SO_4_^2−^, CN^−^, H_2_PO_4_^−^, ACO^−^, N_3_^−^, and NO_2_^−^, only F^−^ induced a significant fluorescence enhancement at 650 nm. This is further corroborated by Figures S7 and S8, where other metal ions and amino acids cause negligible fluorescence changes compared to F^−^, indicating high selectivity for F^−^ against these interferents. Subsequently, for HClO detection in the [DHM + F^−^] system (Figure 3c,d, *λ*_em_ = 460 nm), among species such as HCO_3_^−^, SO_3_^2−^, NO_3_^−^, ^−^O_2_, TBHP, ROO·, ·OH, ACO^−^, Br^−^, Cl^−^, I^−^, Cys, Hcy, and Glutathione, only HClO triggered a prominent fluorescence response at 460 nm. The selectivity toward HClO is also evident in Figure S9 (metal ion interference test) and Figure S10 (amino acid interference test), where only HClO induces a significant fluorescence change while other species show negligible effects. The presence of other interfering substances caused negligible fluorescence changes in both channels. These results demonstrate that DHM exhibits sequential recognition of F^−^ and HClO.

### 3.5 Sensing mechanism study

**FIGURE 4:**
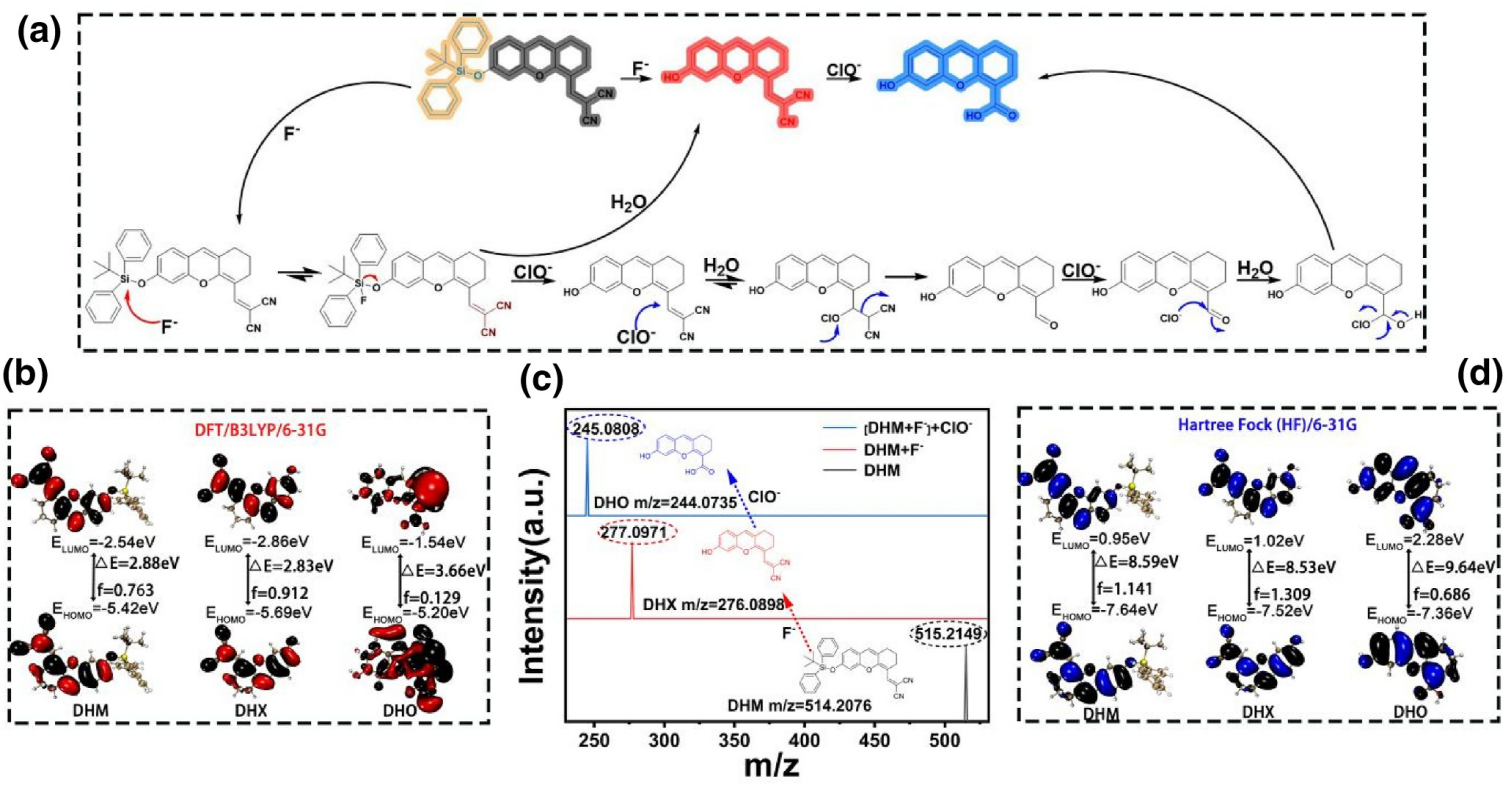
Schematic diagram of the detection mechanism of F^−^ and HClO by DHM (a). Density functional theory and Hartree–Fock calculations for DHM, DHX, and DHO (b, d). HR‐MS of DHM, DHX, and DHO, showing DHM, DHX formed by reaction with F^−^, and DHO formed by further reaction of [DHM + F^−^] with HClO (c). HClO, hypochlorous acid; HR‐MS: high‐resolution mass spectrometry.

**FIGURE 5:**
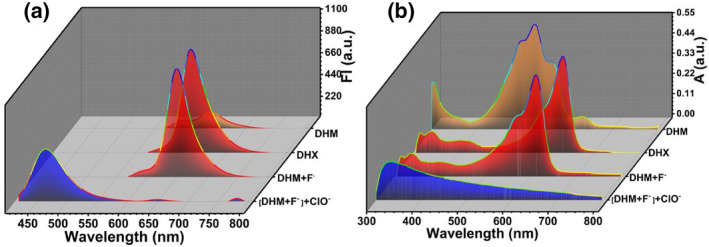
The UV‐Vis absorption of the probe in distinct systems (DHM, DHX, DHM + F^−^, and DHM + F^−^ + HClO) (a); and its fluorescence emission in the same set of systems (b). Data are presented as mean ± SD (*n* = 3).

The sequential detection mechanism of probe DHM towards F^−^ and HClO was thoroughly investigated via a combination of Fourier transform infrared spectroscopy (FT‐IR), high‐resolution mass spectrometry (HRMS), density functional theory (DFT) calculations, structural analysis, and optical property characterization, with key results presented in Figures 4 and 5 and Video S1.

HRMS (ESI^+^) analysis corroborated the sequential molecular transformations of DHM during the cascade detection: the parent probe DHM exhibited an observed mass peak at 515.2149, matching the calculated mass of 515.2076 for the [M + H]^+^ ion (C_33_H_31_N_2_ O_2_ Si^+^) and confirming its structural integrity; upon F^−^ addition, a new peak at 277.0971 was detected, consistent with the calculated mass of 277.0898 for the [M + H]^+^ ion of intermediate DHX (C_17_H_18_N_2_O_2_^+^), verifying DHX formation via F^−^‐induced Si‐O bond cleavage[20, 35]; Subsequent HClO oxidation[^23^, ^43^] resulted in the appearance of a peak at 245.0808, aligning with the calculated mass of 245.0735 for the [M + H]^+^ ion of the final product DHO (C_14_H_13_ O_4_^+^), thus confirming DHO generation (Figure 4c). Complementing these mass spectral data. The FT‐IR spectra of DHM, [DHM + F^−^], DHX, [DHM + F^−^ + HClO], and DHO are illustrated in Figure S18, providing unambiguous spectroscopic evidence for the proposed stepwise sensing cascade key vibrational markers track the progressive molecular changes, with the Si‐O stretching band (1112.4 cm^−1^) a diagnostic feature of the parent DHM‐attenuated in both [DHM + F^−^] and DHX (consistent with F^−^‐triggered Si‐O bond cleavage, confirming DHX as a close structural analog of the [DHM + F^−^] intermediate), while a distinct C≡N stretching peak (2206.2 cm^−1^) emerges in DHX, exhibits reduced intensity in [DHM + F^−^] + HClO, and is entirely absent in DHO‐directly corroborating the HClO‐mediated oxidation of the F^−^‐derived intermediate (encompassing DHX and [DHM + F^−^]) to form DHO. Collectively, these HRMS data and FT‐IR spectral trends, together with supplementary results (Figures S15–S17), unequivocally validate the sequential formation of DHX and DHO through specific reactions with F^−^ and HClO, respectively, providing compelling experimental support for the proposed stepwise sensing mechanism.

The optical properties of DHM across distinct systems (DHM alone, DHX, DHM + F^−^, and [DHM + F^−^] + HClO) are systematically elucidated in Figure 5, while Video S1 provides real‐time visual validation of the sequential sensing process, collectively offering mechanistic insights into its stepwise recognition of F^−^ and HClO. In the UV‐Vis absorption spectra (Figure 5a), DHM exhibits characteristic absorption peaks with a prominent maximum around 520 nm. Upon reaction with F^−^, the [DHM + F^−^] system displays altered absorption peak positions and intensities, with a peak centered at 630 nm‐consistent with the structural features of DHX and reflective of the Si‐O bond cleavage‐driven transformation from DHM to DHX. The [DHM + F^−^] + HClO system further exhibits a distinct absorption profile, with the peak at 650 nm significantly decreased, which is in good agreement with the proposed oxidation of DHX to DHO. Correspondingly, the fluorescence emission spectra (Figure 5b) reveal that DHM has negligible fluorescence, while DHM + F^−^ emits strongly in the red region with a peak around 650 nm and fluorescence intensity similar to that of DHX alone. The [DHM + F^−^] + HClO system presents a shifted emission pattern with the fluorescence maximum at 460 nm, aligning with the formation of DHO with modified electronic conjugation. Notably, Video S1 captures the dynamic color and fluorescence changes during sequential F^−^ and HClO addition: the initially light red DHM solution turns blue with intense red fluorescence upon F^−^ addition (which is indicative of DHX formation), and subsequently reverts to a colorless state with quenched red fluorescence after HClO introduction (presumably associated with the formation of DHO). This real‐time visual evidence directly corroborates the spectral observations in Figure 5 and eliminates ambiguities in the reaction sequence. Collectively, these complementary UV‐Vis, fluorescence, and real‐time video data, together with supplementary evidence, unequivocally validate the stepwise reaction mechanism in which DHM first reacts with F^−^ to form DHX and then DHX reacts with HClO to generate DHO‐each step exhibiting distinct spectral and visual signatures‐thus providing compelling experimental support for the probe's selective and sequential sensing of F^−^ and HClO in complex matrices.

DFT calculations (B3LYP/6‐31G, Figure 4b) further clarified the electronic changes during sensing. For DHM, DHX, and DHO, distinct highest occupied molecular orbital (HOMO) and lowest unoccupied molecular orbital (LUMO) distributions, along with their energy gaps (Δ*E*), were observed: DHM has a Δ*E* of 2.88 eV (−5.42 eV HOMO, −2.54 eV LUMO); DHX shows a slightly reduced Δ*E* of 2.83 eV (−5.69 eV HOMO, −2.86 eV LUMO), consistent with the reshift in fluorescence upon F^−^ binding; DHO exhibits an enlarged Δ*E* of 3.66 eV (−5.20 eV HOMO, −1.54 eV LUMO), aligning with the blue‐shift induced by HClO oxidation. Complementarily, Hartree–Fock (HF)/6‐31G calculations (Figure 4d) also visualized the HOMO and LUMO distributions and their energy gaps for DHM, DHX, and DHO, providing corroborative electronic structure insights. These gap variations directly correlate with the observed spectral shifts, validating that sequential reactions with F^−^ and HClO drive predictable changes in conjugation and electronic properties. Thus, the proposed recognition mechanism is confirmed: F^−^ triggers Si‐O bond cleavage to form DHX, followed by HClO mediated oxidation of DHX to DHO.

In summary, multi‐dimensional experimental (HRMS, FT‐IR, optical characterization) and theoretical (DFT/HF) approaches comprehensively validate that probe DHM detects F^−^ and HClO sequentially via a cascade mechanism: F^−^‐triggered Si‐O bond cleavage forms intermediate DHX (red fluorescence), followed by HClO‐mediated oxidation to DHO (blue‐shifted emission), with HOMO‐LUMO energy gap variations rationalizing the spectral changes.

### 3.6 Determination of F^−^ and HClO in soil, cotton swab, and water samples

**FIGURE 6:**
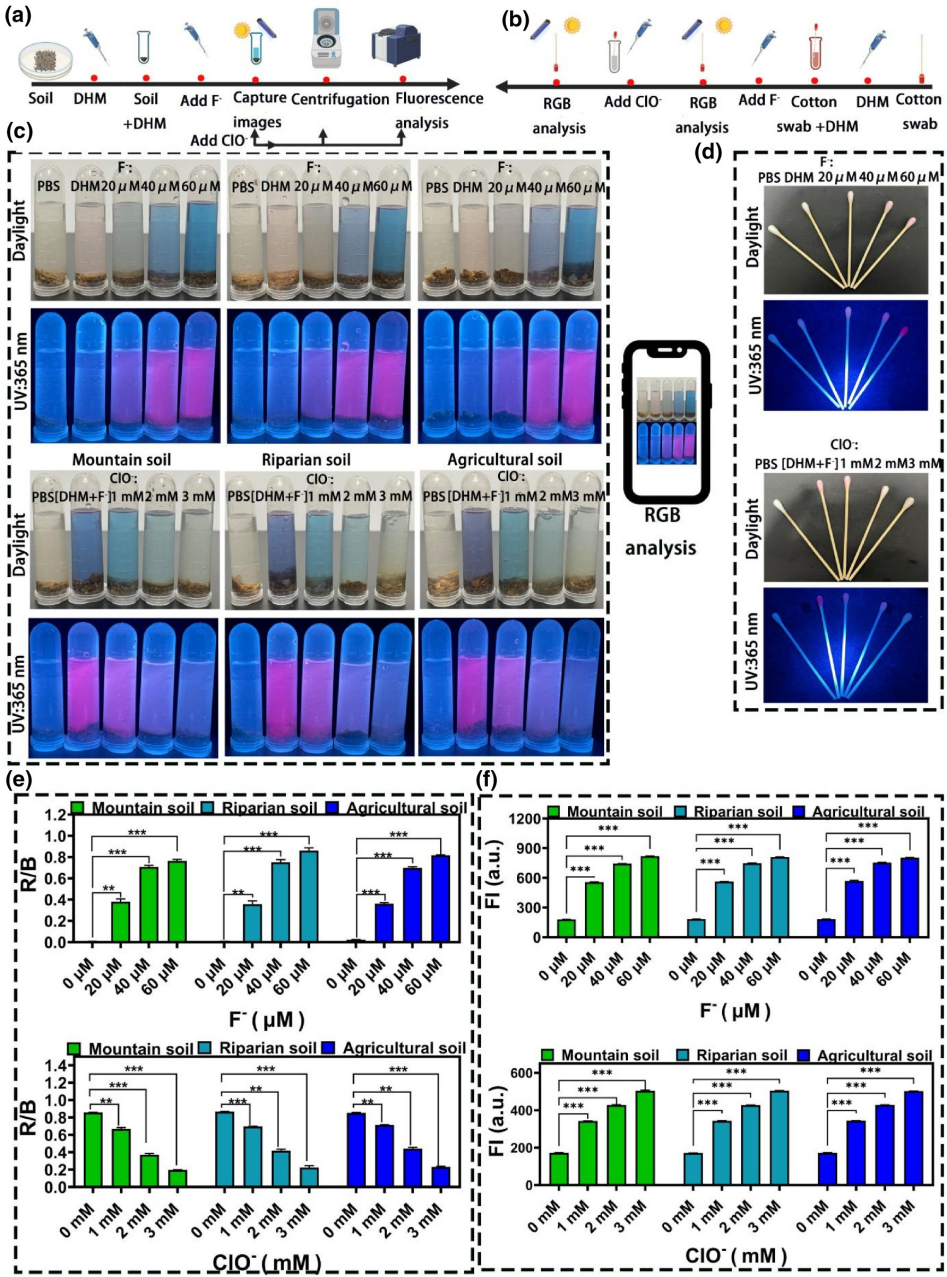
Schematic diagrams of experiments: fluorescence analysis and RGB imaging for probing targets with probe DHM in soil samples (a), and fluorescence imaging and RGB analysis with DHM on a cotton swab (b). Fluorescence images of the DHM probe in soil samples and DHM‐loaded cotton swabs under daylight/UV‐365 nm, upon sequential treatment with F^−^ (0–60 μM) and subsequent addition of HClO (0–3 mM) after introducing 60 μM F^−^ (c, d). Bar graphs of R/B ratios under UV irradiation at 365 nm and fluorescence intensities for soil samples upon sequential treatment with F^−^ (0–60 μM) and HClO (0–3 mM) (e, f). Data are presented as mean ± SD (*n* = 3). *p < 0.03, **p < 0.002, and ***p < 0.001. HClO, hypochlorous acid; RGB, red‐green‐blue.

The practical applicability of DHM as a sequential dualanalyte probe for F^−^ and HClO in complex environmental matrices was evaluated through soil sample and cotton swab‐based assays, as depicted in Figure 6. For soil sample analysis (Figure 6a,c,e,f), DHM was tested in mountain, riparian, and agricultural soils with the sequential addition of F^−^ (0–60 μM) and HClO (0–3 mM) at varying concentrations. Under daylight and UV‐365 nm illumination, distinct sequential fluorescence color changes were observed: F^−^ first induced a red‐shift in fluorescence, followed by a blue‐shift triggered by addition of HClO, consistent with the spectral responses in solution. The bar graphs of B/R ratios and fluorescence intensities showed concentration‐dependent trends across all soil types, with low background interference‐indicating DHM ability to sequentially sense F^−^ and HClO even in matrix‐rich soil environments. These results demonstrate DHM potential for on‐site environmental monitoring of F^−^ and HClO via sequential recognition in soil, where their presence can impact soil health and crop growth.

The cotton swab‐based assay (Figure 6b,d) further highlights the utility of DHM for portable sequential sensing. As quantified by RGB analysis in Figure S19a (for F^−^) and Figure S19b (for HClO), DHM‐loaded cotton swabs exhibited sequential changes in the R/B ratio under UV‐365 nm: an initial significant increase with red fluorescence gradually enhancing upon exposure to F^−^ (60 μM), followed by a subsequent distinct change with red fluorescence gradually weakening when further exposed to HClO (3 mM), with statistically significant differences compared to blank samples. These sequential changes were accompanied by clear fluorescence and color transitions that could be visually discerned. This format offers a user friendly, low‐cost approach for rapid sequential screening of F^−^ and HClO in field settings, eliminating the need for sophisticated instrumentation.

The probe DHM was utilized for the sequential detection of F^−^ and HClO in real water samples to assess its practical applicability (Tables S1 and S2). Three types of real water samples (tap water, river water, and spring water) were first spiked with F^−^, and after confirming F^−^ detection via DHM, the samples were further spiked with HClO. Each real water sample was analyzed in triplicate (*n* = 3). For F^−^ detection (Table S1), the recovered concentrations were consistent with the spiked amounts, with recoveries ranging from 99.12% to 101.30%. For HClO detection (Table S2), subsequent to the pre‐addition of F^−^ and its specific binding with DHM, the recovered HClO concentrations also matched the spiked levels, with recoveries ranging from 99.16% to 100.14%. These results demonstrated that DHM can be applied for the reliable sequential detection of F^−^ and HClO in real aqueous samples.

### 3.7 Determination of F^−^ and HClO in food samples

**FIGURE 7:**
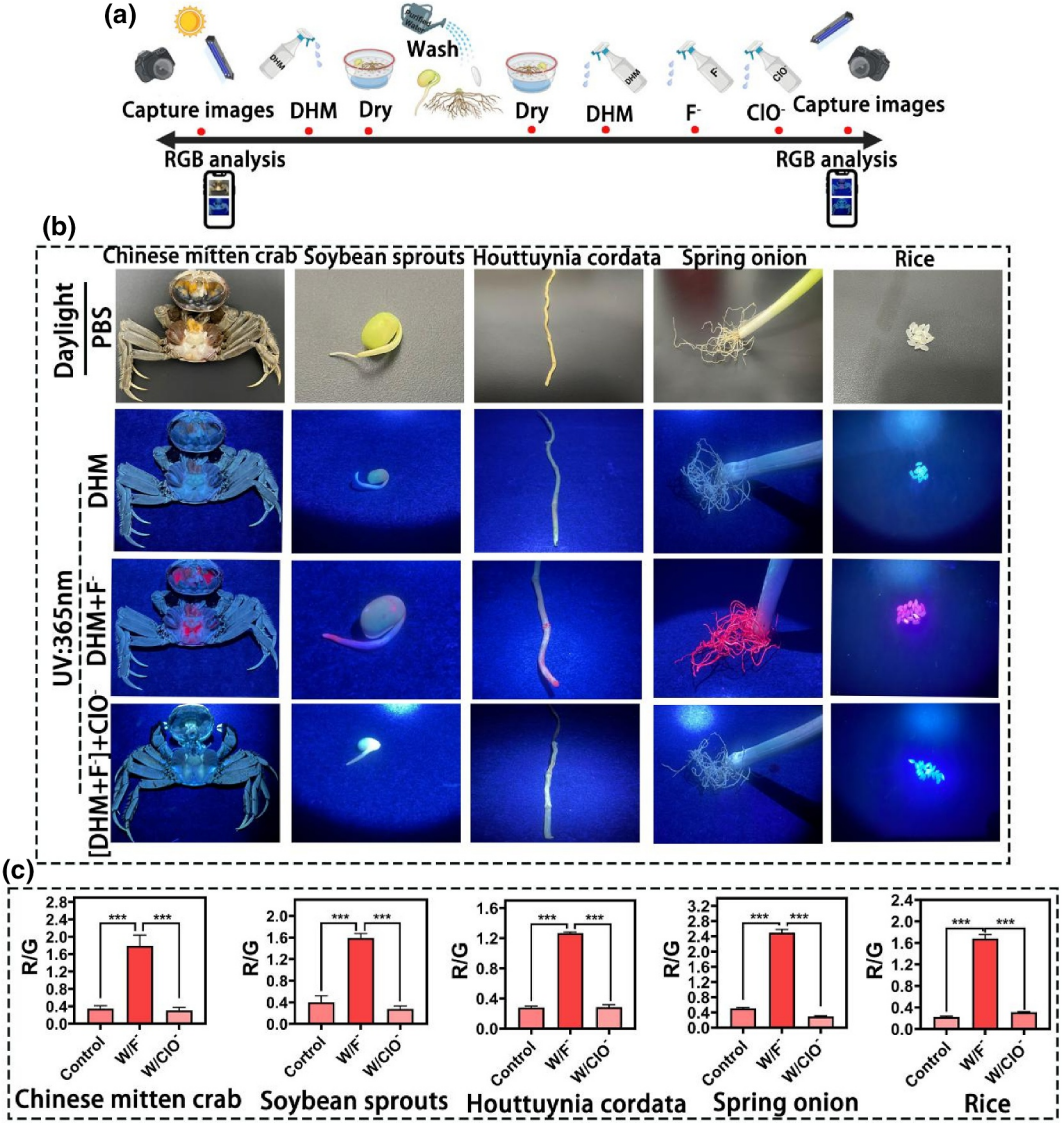
Schematic diagram of a plant imaging and RGB analysis experiment (a). Fluorescence images of various samples (Chinese mitten crab, soybean sprouts, etc.) under daylight/UV‐365 nm, treated with DHM, DHM + F^−^, and DHM + F^−^ + HClO (b). Bar graphs of R/G ratios for Chinese mitten crabs, soybean sprouts, etc., under Control (DHM), W/F^−^ (DHM + F^−^), and W/HClO (DHM + F^−^ + HClO) conditions (c). Data are presented as mean ± SD (*n* = 3). ***p < 0.001. HClO, hypochlorous acid.

To assess the applicability of DHM in biological and food matrices, its performance was evaluated in diverse samples (Chinese mitten crab, soybean sprouts, *H. cordata*, spring onion, and rice) via fluorescence imaging and RGB analysis, as shown in Figure 7. Under daylight and UV‐365 nm illumination (Figure 7b), DHM‐treated samples exhibited distinct fluorescence responses upon sequential F^−^ and HClO addition. Chinese mitten crab, soybean sprouts, and other samples showed negligible fluorescence with DHM alone; F^−^ induced a distinct red fluorescence, while subsequent HClO addition led to the weakening of red fluorescence. These fluorescence changes were consistent with the probe's solution‐phase behavior, demonstrating that DHM can sense F^−^ and HClO in complex biological/food matrices without significant matrix interference. RGB analysis (Figure 7c) quantified these responses via R/G ratio changes. In all samples, the R/G ratio was significantly elevated in the “W/F^−^” (DHM + F^−^) group compared to the control (DHM) and “W/HClO” (DHM + F^−^ + HClO) groups, aligning with the visual fluorescence trends (red fluorescence enhancement upon F^−^ addition and its attenuation with HClO). This consistency confirms that the DHM sensing mechanism (F^−^‐induced silicon–oxygen bond cleavage followed by HClO mediated oxidation) operates effectively in biological/food matrices, enabling selective and sequential detection of F^−^ and HClO.

### 3.8 Live cell imaging

**FIGURE 8:**
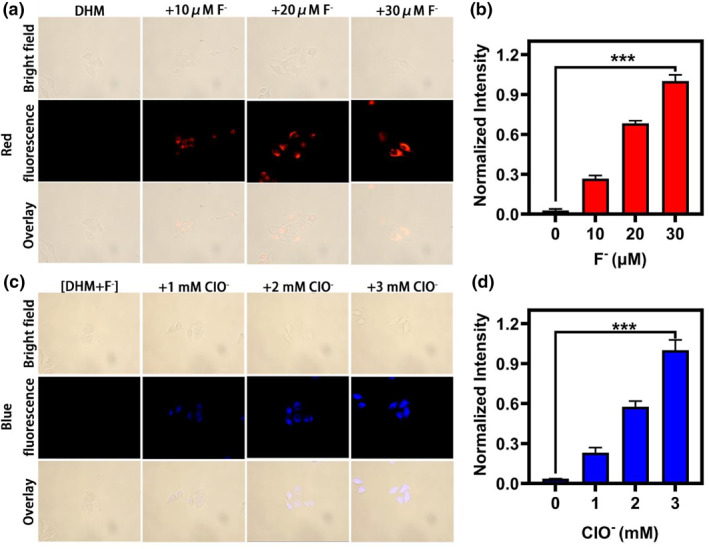
Bright‐field, red/blue fluorescence, and overlay images of cells treated with DHM, then incubated with F^−^ (0–30 μM, a) or [DHM + F^−^] followed by HClO (0–3 mM, c); fluorescence intensity changes depending on F^−^ (b) and HClO (d) concentrations. Data are presented as mean ± SD (*n* = 3). ***p < 0.001. HClO, hypochlorous acid.

Encouraged by the favorable sensing performance of probe DHM in vitro, we further carried out fluorescence imaging experiments in living cells. First, a cytotoxicity assessment (Figure S20) indicated that DHM exhibited low cytotoxicity, enabling its application in live‐cell imaging. Then, we utilized DHM to visualize the sequential detection of exogenous F^−^ and HClO in living cells. Cells treated with DHM alone showed negligible red fluorescence (Figure 8a). When incubated with F^−^ (10–30 μM), the cells displayed a concentration‐dependent red fluorescence enhancement (Figure 8a,b). Subsequently, cells pretreated with DHM and F^−^ were exposed to HClO (1–3 mM), and a distinct blue fluorescence signal was observed, with its intensity increasing with HClO concentration (Figure 8c,d). These results demonstrate that DHM can sequentially detect F^−^ and HClO in living cells, holding great potential for studying the biological roles of these species in cellular environments.

### 3.9 Fluorescence imaging of F^−^ and HClO in mouse

**FIGURE 9:**
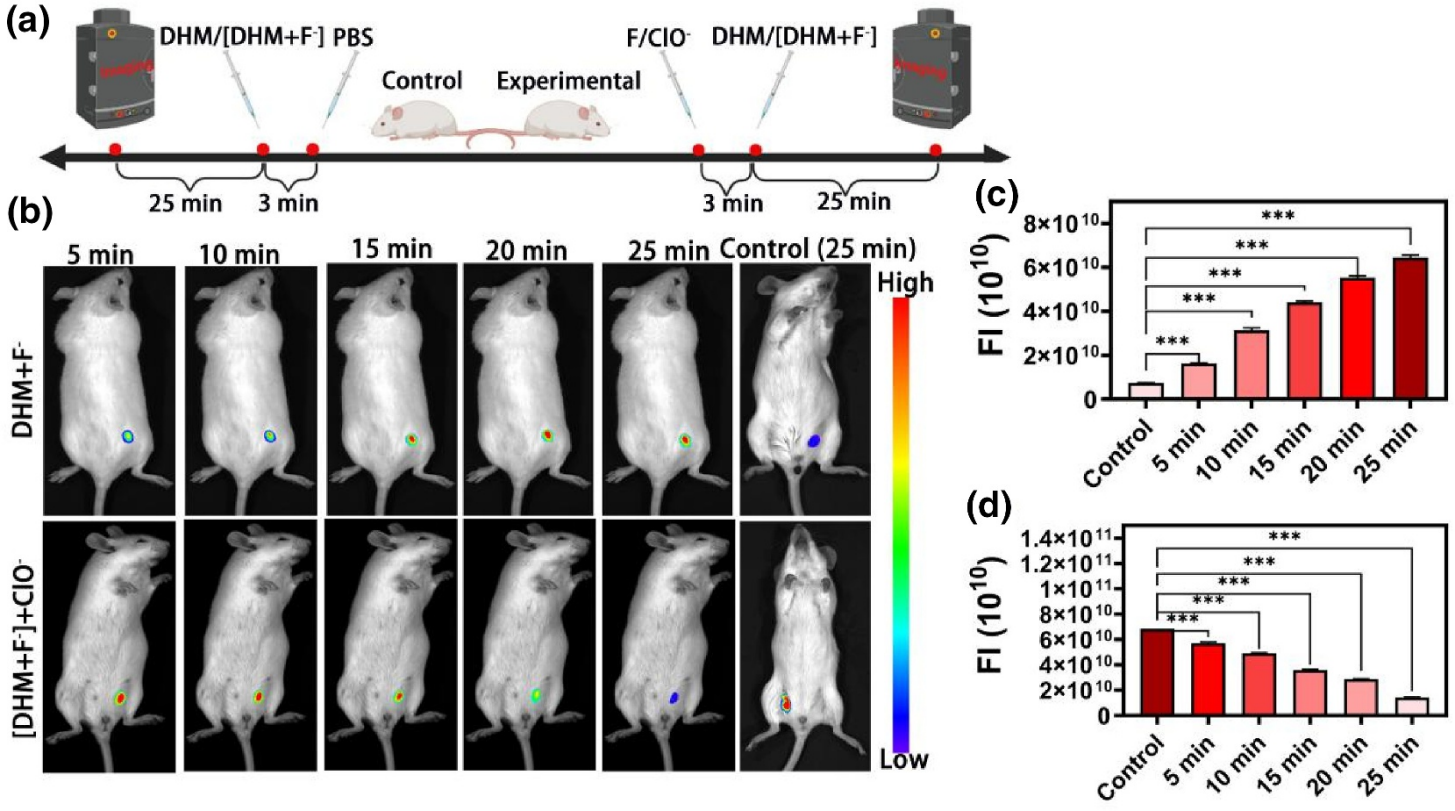
Schematic illustration of in vivo imaging workflow: mice injected with DHM/DHM + F^−^, then F^−^/HClO, followed by imaging (a). In vivo fluorescence images (b) and intensity graphs (c, d) of mice treated with DHM + F^−^ and [DHM + F^−^] + HClO, showing time‐dependent signal changes. Data are presented as mean ± SD (*n* = 3). ***p < 0.001. HClO, hypochlorous acid.

To comprehensively validate DHM's applicability for sequential F^−^ and HClO detection, both in vitro and in vivo studies were conducted: in vitro experiments (Figure S21) clearly demonstrate distinct fluorescence responses, with DHM exhibiting negligible fluorescence, DHM + F^−^ showing a significant enhancement, and [DHM + F^−^] + HClO reverting to low fluorescence; building on this, in vivo fluorescence imaging in mice (Figure 9) further confirmed the probe's performance, where the imaging workflow (Figure 9a) outlined the experimental design involving probe administration and sequential anion exposure, mice administered DHM + F^−^ exhibited time‐dependent red fluorescence enhancement (Figure 9b,c) consistent with in vitro results (confirming effective F^−^ sensing in complex biological environments), and subsequent HClO injection induced a time‐dependent attenuation of red fluorescence (Figure 9b,d), validating sequential detection capability with minimal matrix interference. These findings, coupled with implied low toxicity, highlight DHM potential for studying F^−^ and HClO dynamics in physiological contexts, bridging fundamental sensing research and biomedical applications.

## 4 CONCLUSION

In summary, we developed a novel dual‐response fluorescent probe (DHM) based on a dihydroxanthene moiety for sequential, discriminative detection of F^−^ and HClO across biological, environmental, and food matrices. F^−^ induces red fluorescence at 650 nm (LOD = 14 nM) via silicon–oxygen bond cleavage, while HClO triggers blue fluorescence at 460 nm (LOD = 3.19 μM) through oxidation (190 nm spectral separation eliminates crosstalk). It exhibits rapid kinetics (20 s for F^−^, 3 min for HClO), robust selectivity, and low cytotoxicity, supporting Huh7 cell imaging, in vivo mouse imaging, and reliable quantitative detection in real samples (soil, water, food, cotton swabs) with recoveries of 99.12%–101.30% (F^−^) and 99.16%–100.14% (HClO), offering a practical tool for F^−^/HClO monitoring and a blueprint for dual‐analyte probe design.

## CONFLICT OF INTEREST STATEMENT

The authors declare no conflicts of interest.

## ETHICS STATEMENT

All animal experiments conducted in this study were performed in strict accordance with the guidelines established by the Animal Ethics Care Committee of Jinzhou Medical University, with the approval of Protocol No. JZMULL‐2025‐382.

## Supporting information

Supporting Information S1

Video S1

## Data Availability

The data that support the findings of this study are available upon request from the corresponding author. The data are not publicly available due to privacy or ethical restrictions.
