# Supplementary material for: A multifunctional near‐infrared dual‐channel off‐on‐off probe for sequential detection of fluoride and hypochlorous acid: Multimatrix applications
Source: Smart Mol. 2026 Aug 17:e70092. Online ahead of print. doi: 10.1002/smo2.70092 (PMC13481731; doi:10.1002/smo2.70092)
Supplement: Supplementary file 1 [file SMO2-9999-0-s001.docx]

**Supplementary Material**

**A Multifunctional Near-Infrared Dual-Channel Off-On-Off Probe for Sequential Detection of Fluoride and Hypochlorous Acid: Multimatrix Applications**

Tongtong Li^1^ , Xiaodong Zhang^2*^, Tong Yu^1^ , Dong Cai^1^ , Yabai Li^3^ , Shiyu Wu^2^ , Jingyi Gao^2^ , Yaqian Xu^2^ , Yihui Sun^2^ , Xuewei Pu^1*^, Fanjie Meng^1*^

*^*^ Corresponding author (E-mail: zhangxddlut@163.com (Xiaodong Zhang), xueweipu@126.com (Xuewei Pu ), mengfj@jzmu.edu.cn (Fanjie Meng)*

^1^College of Pharmacy, Jinzhou Medical University, JinZhou, 121000 , China

^2^Linyi Vocational University of Science and Technology, LinYi, 276000, China

^3^School of Chemical Engineering, Shandong Institute of Petroleum and Chemical Technology, DongYing, 257000, China

**Table of contents**

**[1. List of figures and tables	2](#_Toc24479)**

**[2. Experimental section	3](#_Toc30297)**

**[3. Copy of NMR and Mass for the compounds	1](#_Toc11451)1**

**[4. Crystal structures and data:	1](#_Toc17038)6**

**[5. References	2](#_Toc7196)5**

1. **List of Figures and Tables**

**[Fig. S1.](#_Toc10663)** [Fluorescence emission spectra of probe DHM in various solvents with and without F](#_Toc10663)^[-](#_Toc10663)^	[7](#_Toc10663)

**[Fig. S2.](#_Toc14562)** [Fluorescence spectral responses of probe DHM (with and without F](#_Toc14562)^[-](#_Toc14562)^ [) to water fraction in DMSO-water system	8](#_Toc14562)

**[Fig. S3.](#_Toc10249)** [Time-dependent fluorescence response of probe DHM (with and without F](#_Toc10249)^[-](#_Toc10249)^[) within 60 minutes	8](#_Toc10249)

**[Fig. S4.](#_Toc22913)** [pH-dependent fluorescence responses of probe DHM (with and without F](#_Toc22913)^[-](#_Toc22913)^[) in aqueous solution	9](#_Toc22913)

**[Fig. S5.](#_Toc2051)** [Time-dependent fluorescence response of [DHM+F](#_Toc2051)^[-](#_Toc2051)^[] complex upon addition of HClO	9](#_Toc2051)

**[Fig. S6.](#_Toc13482)** [Fluorescence imaging of samples in centrifuge tubes under UV illumination.	10](#_Toc13482)

**[Fig. S7.](#_Toc17245)** [Fluorescence responses of various metal ions and F](#_Toc17245)^[-](#_Toc17245)^[.	10](#_Toc17245)

**[Fig. S8.](#_Toc25023)** [Fluorescence responses of amino acids and F](#_Toc25023)^[-](#_Toc25023)^[.	11](#_Toc25023)

**[Fig. S9.](#_Toc19931)** [Fluorescence responses of various metal ions and HClO.	11](#_Toc19931)

**[Fig. S10.](#_Toc32688)** [Fluorescence responses of amino acids and HClO.	12](#_Toc32688)

**[Fig. S11.](#_Toc25572)** ^[1](#_Toc25572)^[H NMR spectrum of DHM in CDCl](#_Toc25572)_[3](#_Toc25572)_[.	12](#_Toc25572)

**[Fig. S12.](#_Toc17196)** ^[13](#_Toc17196)^[C NMR spectrum of DHM in CDCl](#_Toc17196)_[3](#_Toc17196)_[.	13](#_Toc17196)

**[Fig. S13.](#_Toc22859)** ^[1](#_Toc22859)^[H NMR spectrum of DHX in DMSO-d6.	13](#_Toc22859)

**[Fig. S14.](#_Toc17834)** ^[13](#_Toc17834)^[C NMR spectrum of DHX in DMSO-d6.	14](#_Toc17834)

**[Fig. S15.](#_Toc13000)** [Electrospray ionization mass spectrum of DHM.	14](#_Toc13000)

**[Fig. S16.](#_Toc22898)** [Electrospray ionization mass spectrum of DHM+F](#_Toc22898)^[-](#_Toc22898)^[.	15](#_Toc22898)

**[Fig. S17.](#_Toc19800)** [Electrospray ionization mass spectrum of [DHM+F](#_Toc19800)^[-](#_Toc19800)^[]+HClO.	15](#_Toc19800)

**[Fig. S18.](#_Toc25639)** [FT-IR spectra of DHM, DHM+F, DHX, [DHM+F](#_Toc25639)^[-](#_Toc25639)^[]+HClO, and DHO, with characteristic peaks of C≡N and Si-O.	15](#_Toc25639)

**[Fig. S19.](#_Toc2469)** [RGB data plots of the probe on cotton swabs after addition of F](#_Toc2469)^[-](#_Toc2469)^ [(a) and sequential addition of F](#_Toc2469)^[-](#_Toc2469)^ [followed by HClO (b).	15](#_Toc2469)

**[Fig. S20.](#_Toc22299)** [Cytotoxicity of probe DHM towards Huh7 cells.	15](#_Toc22299)

**[Fig. S21.](#_Toc476)** [Fluorescence Imaging and Intensity Analysis of Probe DHM upon Sequential Addition of F](#_Toc476)^[-](#_Toc476)^ [and HClO .	15](#_Toc476)

**Video S1.** Dynamic fluorescence imaging video of DHM probe.	16

**[Table S1.](#_Toc8540)** [Determination of F](#_Toc8540)^[-](#_Toc8540)^ [in real water samples using probe DHM.	16](#_Toc8540)

**[Table S2.](#_Toc6150)** [Determination of HClO in real water samples using probe [DHM+F](#_Toc6150)^[-](#_Toc6150)^[].	17](#_Toc6150)

**[Table S3.](#_Toc22332)** [DHM crystal’s selected bond lengths (Å), bond angles (°), and torsion angles (°) obtained from experimental (XRD) methods.	18](#_Toc22332)

**[Table S4.](#_Toc2027)** [Comparison of single-function probes for detecting F](#_Toc2027)^[-](#_Toc2027)^[.	2](#_Toc2027)0

**[Table S5.](#_Toc25374)** [Comparison of single-function probes for detecting HClO.	22](#_Toc25374)

**[Table S6.](#_Toc8503)** [Comparison of dual-function probes for detecting F](#_Toc8503)^[-](#_Toc8503)^ [and HClO.	24](#_Toc8503)

**2.Experimental section**

**2.1 Materials and Instruments**

¹H NMR and ¹³C NMR spectra were recorded on a Bruker DMX-600 spectrometer with samples dissolved in CDCl_3_ and DMSO-*d*_6_. High-resolution mass spectra (HRMS) were acquired using an Agilent Q-TOF 6510 mass spectrometer. Fourier transform infrared (FT-IR) spectra were measured on a Shimadzu IRAffinity-1 spectrophotometer. UV-visible absorption and fluorescence spectra were measured with a Hitachi U-3010 spectrophotometer and F-4700 fluorometer, respectively. Fluorescent images of cells were acquired using a Zeiss Axio Vert A1 inverted fluorescence microscope. In vivo animal imaging was performed using the BLT AniView600 SE multimodal in vivo imaging system.Thin-layer chromatography (TLC) analysis was conducted on silica gel plates, and column chromatography was performed using silica gel (300–400 mesh). Both TLC plates and silica gel were purchased from Qingdao Ocean Chemicals.

**2.2 Synthesis of DHM.**

**Scheme. S1.** Synthesis of DHM.

Compound DHX was synthesized depending on a modified procedure of the reported literature[1]. Briefly, DHX (0.29 mmol, 80.0 mg), tert-Butyldiphenylchlorosilane (0.35 mmol, 96.0 mg) and TEA (100 uL) were added into a three-neck round-bottom flask and completely dissolved in DCM (10 mL). The above mixture was stirring at room temperature under N_2_ atmosphere. After 4 h, the solvent was removed under reducing pressure to give a residue. Finally, the crude product was purified by silica column chromatography using DCM/PE as the eluent 5/1 = v/v).The target compound was obtained as a Red solid with a yield of 89%. DHX: ^1^H NMR (600 MHz, DMSO-d6) δ 10.55 (s, 1H), 8.00 (s, 1H), 7.29 (d, J = 8.5 Hz, 1H), 7.23 (s, 1H), 6.83 (s, 1H), 6.72 (s, 1H), 2.67 (s, 2H), 2.52 (s, 2H), 1.71 (s, 2H). ^13^C NMR (150 MHz, DMSO-d6) δ 160.91, 159.20, 153.69, 149.44, 132.70, 128.63, 124.44, 117.79, 116.33, 113.56, 108.80, 102.11, 66.28, 27.99, 24.23, 20.00. DHM: ^1^H NMR (400 MHz, CDCl_3_) δ 7.85 (s, 1H), 7.67-7.62 (m, 4H), 7.43-7.37 (m, 2H), 7.33 (dd, J = 7.9, 6.4 Hz, 4H), 6.90 (d, J = 8.5 Hz, 1H), 6.74 (s, 1H), 6.59 (d, J = 2.3 Hz, 1H), 6.48 (dd, J = 8.4, 2.3 Hz, 1H), 2.76 (t, J = 6.1 Hz, 2H), 2.51 -2.45 (m, 2H), 1.72 (p, J = 6.2 Hz, 2H), 1.04 (s, 9H). ^13^C NMR (101 MHz, CDCl_3_) δ 158.48 , 150.41 , 135.43 , 131.85 , 130.65 , 130.36 , 128.07 , 127.60 , 117.35 , 109.85 , 107.05 , 77.36 , 29.16 , 26.39 , 24.76 , 20.48 , 19.50. HRMS (ESI): m/z [M+H]^+^ calcd. for [DHM]^+^: 515.2076, found: 515.2159.

**2.3 Crystal preparation and testing methods of DHM**

A total of 40 mg of DHM was dissolved in a mixed solvent of petroleum ether and Dichloromethane (3:1, v/v). Red block-shaped crystals were obtained via slow solvent evaporation. The crystal structure of DHM was determined using a Rigaku Goni X single-crystal diffractometer.

**2.4 Absorption and fluorescence spectra measurements**

DHM (5.15 mg) was dissolved in DMSO (10.0 mL) to prepare a 1.0 mM stock solution and stored in the refrigerator at 4℃and keeped dark. Stock solutions of relevant analytes-including anions (F^-^ , Br^-^ , Cl^-^ , I^-^ , SCN^-^ , NO_3_^-^ , ClO_4_^-^, HCO_3_^-^ , HSO_3_^-^ , CO_3_^2-^ , SO_4_^2-^, CN^-^ , H_2_PO_4_^-^ , ACO^-^ , N_3_^-^ , NO_2_^-^, O_2_^-^ , TBHP, ROO·, ·OH) , amino acids (Val, Glu, Trp, Pro, Leu, Thr, Arg, Phe, Gly, Cys, Hcy, GSH), and metal ions (Mg²⁺, Al³⁺, Cu²⁺, Zn²⁺, Na⁺, Cu⁺, K⁺, Cs⁺, Li⁺, Cr³⁺, Ni²⁺, Fe³⁺, Sn²⁺, Cd²⁺, Pd²⁺, Fe²⁺, Ag⁺, Co²⁺, Hg²⁺) were prepared by dissolving them in ultrapure water. For polarity-sensing studies, DHM (10.0 μM) was dissolved in solvents of varying polarities, including dimethyl sulfoxide (DMSO), tetrahydrofuran (THF), ethanol, acetonitrile, IPA, PBS, and DMSO-water mixed solvents with different volume ratios; absorption and fluorescence spectra were then measured. In F^-^-sensing studies, spectra were recorded in aqueous media. For titration experiments, DHM (5.0 μM) was mixed with F^-^ at concentrations ranging from 0.0 to 120.0 μM. For selectivity and anti-interference experiments, DHM (5.0 μM) was mixed with either F^-^ (100.0 μM) or other relevant analytes (100.0 μM).a subsequent titration experiment with HClO was conducted, where the concentration of HClO varied from 0.0 to 5 mM. For selectivity and anti-interference experiments, DHM (5.0 μM) was mixed with either F^-^ (100.0 μM), HClO (5 mM) or other relevant analytes (5 mM). All measurements were performed at room temperature (25-28°C).

**2.5 Theoretical calculations**

To investigate the mechanism underlying DHM’s polarity-sensing behavior, computational calculations were performed based on density functional theory (DFT). The Chem3D, Gaussian 09 W, VMD, Multiwfn, and GaussView 5.0 programs were employed for this purpose. Calculations of the highest occupied molecular orbital (HOMO) and the lowest unoccupied molecular orbital (LUMO) were carried out to elucidate the optical properties of DHM.

**2.6 Fluorescence imaging of F^-^ and HClO in Real samples**

Water samples were collected from different types of water bodies, and soil samples were collected from various soil types. The collection of all samples followed legal protocols. The samples were stored in sealed, light-protected containers and used promptly after collection. Water samples were filtered through filter paper and directly subjected to analysis. For soil samples, they were mixed with relevant solutions, centrifuged, and filtered. The standard addition method was utilized for the determination of analytes: a fixed concentration of the target substance was added, and the fluorescence signal was measured using a fluorescence spectrophotometer. Additionally, a smartphone app was employed for RGB analysis to assist in the determination. The analyte concentration was then determined by comparing the signal to a standard curve, enabling the calculation of recovery rates. For specific experiments involving cotton swabs (as a sampling or detection medium), DHM was loaded onto cotton swabs, which were then used to interact with analytes. Soil samples were divided into control and experimental groups, treated with relevant reagents respectively. After treatment, the soils were processed via centrifugation and filtration, and the resulting solution was introduced into the probe system. The fluorescence signals were measured using a fluorescence spectrophotometer, and the smartphone app was also used for RGB analysis to determine the presence and concentration of the target analyte, with observations made under ultraviolet light (365 nm) and natural light. Meanwhile, cotton swabs loaded with DHM were employed for visual and spectral analysis of analytes, with color changes observed under daylight and UV 365 nm light.

**2.7 Fluorescence imaging of F^-^ and HClO in Real Food Samples**

Food samples including rice, Chinese mitten crab, soybean sprouts, Houttuynia cordata, and spring onion were procured from a local morning market. After appropriate treatment, the samples were exposed to 10 μM DHM probe solution. Subsequently, 100 μM F^-^ and 3 mM HClO target analyte solutions were sequentially applied to the surfaces of the food samples. The presence of analytes was monitored and imaged under daylight and a 365 nm UV lamp; images were captured using a Fujifilm XT5 camera under standardized parameters (fixed aperture, exposure time, and ISO) to ensure consistency across samples, and the acquired images were subsequently subjected to RGB analysis via a smartphone app.

**2.8 Cellular imaging**

Huh7 cells were maintained in Dulbecco's Modified Eagle Medium (DMEM) supplemented with 10% fetal bovine serum (FBS) and cultured in a humidified incubator at 37℃ under a 5% CO_2_ atmosphere. The cytotoxicity of the probe was assessed using the Cell Counting Kit-8 (CCK-8) assay. Briefly, Huh7 cells were seeded in 96-well plates and allowed to adhere overnight. Subsequently, the cells were treated with various concentrations of DHM (0, 5, 10, 15, and 20 μM) for 12 hours. After the treatment period, CCK-8 reagent was added to each well, and the absorbance was measured at a wavelength of 450 nm using a microplate reader to determine the cell viability.

To evaluate the performance of the DHM probe for detecting F^-^ and HClO in living cells, Huh7 cells were cultured in complete medium at 37℃ with 5% CO_2_ until reaching 80% confluence. The Huh7 cells were first incubated with 5.0 μM DHM probe for 30 minutes at 37℃, followed by washing with phosphate-buffered saline (PBS) to remove excess probe. For F^-^ detection, the Huh7 cells were then treated with F^-^ at concentrations of 0, 10, 20, and 30 μM; for HClO detection, the Huh7 cells were first incubated with [DHM+F^-^] and then treated with HClO at concentrations of 0, 1, 2, and 3 mM. Fluorescence imaging was performed using a fluorescence microscope equipped with appropriate filter sets: the red channel, designated for F^-^ detection, utilized an excitation wavelength of 530 nm and collected emission wavelengths in the window of 650 nm, while the blue channel, for HClO detection, employed an excitation wavelength of 380 nm and collected emission wavelengths in the window of 460 nm. Bright-field, red/blue fluorescence, and overlay images were captured, and fluorescence intensity was analyzed.

**2.9 Mouse imaging**

To evaluate the in vivo performance of the DHM probe for detecting F^-^ and HClO, all animal experiments conducted in this study were performed in strict accordance with the guidelines established by the Animal Ethics Care Committee of Jinzhou Medical University, with the approval of Protocol No. JZMULL-2025-382. Mice were divided into three groups: the first two groups received subcutaneous injections of F^-^ and HClO (as the experimental groups) respectively, while the third group was given a subcutaneous injection of PBS (as the control group), with all injections administered at designated experimental sites. After the initial injections, the DHM/[DHM+F^-^] probe was uniformly administered via subcutaneous injection at the same sites in all three groups to ensure sufficient contact between the probe and target analytes (or PBS for the control). A 25-minute incubation period was allowed to facilitate probe-analyte interaction, followed by a 3-minute standing period to stabilize the experimental state. Subsequently, fluorescence imaging was performed at 5, 10, 15, 20, and 25 min post-probe administration using an in vivo fluorescence imaging system with the excitation wavelength set at 465 nm and the emission wavelength at 680 nm. Fluorescence intensity was analyzed for both F^-^ and HClO detection across the specified time points, with the PBS group data used to verify the specificity of the probe response.

 **Fig. S1.** Fluorescence emission spectra of probe DHM in various solvents with and without F^-^.

Fig. S2. Fluorescence spectral responses of probe DHM (with and without F^-^) to water fraction in DMSO-water system

Fig. S3. Time-dependent fluorescence response of probe DHM (with and without F^-^) within 60 minutes

Fig. S4. pH-dependent fluorescence responses of probe DHM (with and without F^-^) in aqueous solution

Fig. S5. Time-dependent fluorescence response of [DHM+F^-^] complex upon addition of HClO.

(a) **(b)**

Fig. S6. Fluorescence imaging of samples in centrifuge tubes under UV illumination. (a): 1, Blank; 2,F^-^; 3,Br^-^; 4,Cl^-^; 5,I^-^; 6,SCN^-^; 7,NO_3_^-^; 8,ClO_4_^-^; 9,HCO_3_^-^; 10,HSO_3_^-^; 11,CO_3_^2-^; 12,SO_4_^2-^; 13,CN^-^; 14,H_2_PO_4_^2-^; 15,ACO^-^; 16,N_3_^-^; 17,NO_2_^-^. (b): 1, HClO; 2, Blank; 3, HCO_3_^-^; 4,SO_4_^2-^; 5,NO_3_^-^; 6,^-^O_2_; 7,TBHP; 8,ROO· ; 9, ·OH; 10,ACO^-^; 11,Br^-^; 12,F^-^; 13,Cl^-^; 14,I^-^; 15, Cys; 16, Hcy.

Fig. S7. Fluorescence responses of various metal ions and F^-^.

Fig. S8. Fluorescence responses of amino acids and F^-^.

Fig. S9. Fluorescence responses of various metal ions and HClO.

Fig. S10. Fluorescence responses of amino acids and HClO.

**Fig. S11.** ^1^H NMR spectrum of DHM in CDCl_3_.

**Fig. S12.** ^13^C NMR spectrum of DHM in CDCl_3_.

Fig. S13. ^1^H NMR spectrum of DHX in DMSO-d6.

Fig. S14. ^13^C NMR spectrum of DHX in DMSO-d6.

Fig. S15. Electrospray ionization mass spectrum of DHM.

Fig. S16. Electrospray ionization mass spectrum of DHM+F^-^.

Fig. S17. Electrospray ionization mass spectrum of [DHM+F^-^]+HClO.

Fig. S18. FT-IR spectra of DHM, DHM+F, DHX, [DHM+F^-^]+HClO, and DHO, with characteristic peaks of C≡N and Si-O.

Fig. S19. RGB data plots of the probe on cotton swabs after addition of F^-^ (a) and sequential addition of F^-^ followed by HClO (b).

Fig. S20. Cytotoxicity of probe DHM towards Huh7 cells.

Fig. S21. Fluorescence Imaging and Intensity Analysis of Probe DHM upon Sequential Addition of F^-^ and HClO.

**Video S1.** Dynamic fluorescence imaging video of DHM probe.

Table S1. Determination of F^-^ in real water samples using probe DHM.

| Samples | Found | Added / μM | Found / μM  (n=3) | Recovery (%) | RSD (%) |
| --- | --- | --- | --- | --- | --- |
| Tap water | Not detected | 5 | 5.05±0.05 | 100.93 | 0.99 |
|  |  | 10 | 10.07±0.01 | 100.73 | 0.05 |
| River water | Not detected | 5 | 4.99±0.03 | 99.93 | 0.40 |
|  |  | 10 | 10.13±0.01 | 101.30 | 0.10 |
| Spring water | Not detected | 5 | 4.95±0.02 | 99.12 | 0.30 |
|  |  | 10 | 10.06±0.06 | 100.56 | 0.49 |

Table S2. Determination of HClO in real water samples using probe [DHM+F^-^].

| Samples | Found | Added / μM | Found / μM  (n=3) | Recovery (%) | RSD (%) |
| --- | --- | --- | --- | --- | --- |
| Tap water | Not detected | 100 | 99.39±1.08 | 99.38 | 1.08 |
|  |  | 300 | 300.43±0.73 | 100.14 | 0.24 |
| River water | Not detected | 100 | 99.61±0.60 | 99.61 | 0.61 |
|  |  | 300 | 299.54±0.61 | 99.85 | 0.20 |
| Spring water | Not detected | 100 | 99.17±0.19 | 99.16 | 0.19 |
|  |  | 300 | 298.24±0.68 | 99.41 | 0.23 |

Table S3. DHM crystal’s selected bond lengths (Å), bond angles (°), and torsion angles (°) obtained from experimental (XRD) methods.

| **Bond length (Å)** | | **Bond angles (°)** | |
| --- | --- | --- | --- |
| Atom | length (Å) | Atom | angles (°) |
| Si1-O1 | 1.6611(17) | O1-Si1-C18 | 102.03(10) |
| Si1-C18 | 1.864(3) | O1-Si1-C24 | 107.82(10) |
| Si1-C24 | 1.867(3) | O1-Si1-C30 | 109.57(11) |
| Si1-C30 | 1.884(3) | C18-Si1-C24 | 110.01(11) |
| O2-C5 | 1.378(3) | C18-Si1-C30 | 111.08(12) |
| O2-C7 | 1.370(3) | C24-Si1-C30 | 115.43(13) |
| O1-C1 | 1.363(3) | C7-O2-C5 | 122.15(18) |
| C5-C6 | 1.370(3) | C1-O1-Si1 | 134.28(16) |
| C5-C4 | 1.389(3) | O2-C5-C4 | 120.2(2) |
| C7-C13 | 1.370(3) | C6-C5-O2 | 116.3(2) |
| C7-C8 | 1.433(3) | C6-C5-C4 | 123.5(2) |
| C1-C6 | 1.385(3) | O2-C7-C8 | 118.4(2) |
| C1-C2 | 1.390(3) | C13-C7-O2 | 116.4(2) |
| C13-C14 | 1.416(3) | C13-C7-C8 | 125.2(2) |
| C13-C12 | 1.514(3) | O1-C1-C6 | 117.2(2) |
| C18-C23 | 1.391(3) | O1-C1-C2 | 122.8(2) |
| C18-C19 | 1.393(3) | C6-C1-C2 | 119.9(2) |
| C24-C25 | 1.390(4) | C5-C6-C1 | 118.8(2) |
| C24-C29 | 1.393(4) | C7-C13-C14 | 118.4(2) |
| C4-C3 | 1.403(3) | C7-C13-C12 | 118.8(2) |
| C4-C9 | 1.426(3) | C14-C13-C12 | 122.7(2) |
| C14-C15 | 1.379(4) | C23-C18-Si1 | 121.23(19) |
| C2-C3 | 1.372(3) | C23-C18-C19 | 116.5(2) |
| C12-C11 | 1.494(4) | C19-C18-Si1 | 122.30(19) |
| C15-C16 | 1.431(4) | C25-C24-Si1 | 118.49(19) |
| C15-C17 | 1.428(4) | C25-C24-C29 | 116.8(3) |
| C8-C9 | 1.344(3) | C29-C24-Si1 | 124.6(2) |
| C8-C10 | 1.505(4) | C5-C4-C3 | 116.2(2) |
| C23-C22 | 1.377(4) | C5-C4-C9 | 117.6(2) |
| C19-C20 | 1.386(4) | C3-C4-C9 | 126.1(2) |
| C25-C26 | 1.380(4) | C15-C14-C13 | 130.9(2) |
| C30-C33 | 1.539(4) | C3-C2-C1 | 120.0(2) |
| C30-C31 | 1.541(4) | C2-C3-C4 | 121.7(2) |
| C30-C32 | 1.530(4) | C11-C12-C13 | 112.5(2) |
| C29-C28 | 1.381(4) | C14-C15-C16 | 118.8(3) |
| C16-N1 | 1.142(4) | C14-C15-C17 | 126.5(3) |
| C17-N2 | 1.141(4) | C17-C15-C16 | 114.7(2) |
| C20-C21 | 1.370(4) | C7-C8-C10 | 115.9(2) |
| C21-C22 | 1.364(4) | C9-C8-C7 | 119.3(2) |
| C27-C26 | 1.370(5) | C9-C8-C10 | 124.7(2) |
| C27-C28 | 1.364(5) | C8-C9-C4 | 122.2(2) |
| C10-C11 | 1.423(4) | C22-C23-C18 | 122.2(3) |
|  |  | C20-C19-C18 | 121.4(3) |
|  |  | C26-C25-C24 | 121.9(3) |
|  |  | C33-C30-Si1 | 111.1(2) |
|  |  | C33-C30-C31 | 107.9(3) |
|  |  | C31-C30-Si1 | 107.0(2) |
|  |  | C32-C30-Si1 | 112.5(2) |
|  |  | C32-C30-C33 | 108.8(3) |
|  |  | C32-C30-C31 | 109.4(3) |
|  |  | C28-C29-C24 | 121.4(3) |
|  |  | N1-C16-C15 | 179.2(4) |
|  |  | N2-C17-C15 | 175.5(4) |
|  |  | C21-C20-C19 | 120.0(3) |
|  |  | C22-C21-C20 | 120.0(3) |
|  |  | C21-C22-C23 | 119.9(3) |
|  |  | C28-C27-C26 | 120.4(3) |
|  |  | C27-C26-C25 | 119.5(3) |
|  |  | C27-C28-C29 | 120.0(3) |
|  |  | C11-C10-C8 | 113.7(3) |
|  |  | C10-C11-C12 | 117.2(3) |

| **Torsion Angles(°)** | |
| --- | --- |
| Atom | angles (°) |
| Si1-O1-C1-C6 | 132.1(2) |
| Si1-O1-C1-C2 | -50.2(3) |
| Si1-C18-C23-C22 | -177.8(2) |
| Si1-C18-C19-C20 | 178.5(2) |
| Si1-C24-C25-C26 | 179.5(2) |
| Si1-C24-C29-C28 | -179.2(2) |
| O2-C5-C6-C1 | 179.9(2) |
| O2-C5-C4-C3 | -179.9(2) |
| O2-C5-C4-C9 | -2.3(4) |
| O2-C7-C13-C14 | 1.1(3) |
| O2-C7-C13-C12 | 178.0(2) |
| O2-C7-C8-C9 | -1.3(4) |
| O2-C7-C8-C10 | 176.6(3) |
| O1 Si1 C18 C23 | -159.9(2) |
| O1-Si1-C18-C19 | 21.6(2) |
| O1-Si1-C24-C25 | 26.2(2) |
| O1-Si1-C24-C29 | -155.8(2) |
| O1-Si1-C30-C33 | -166.3(2) |
| O1-Si1-C30-C31 | -48.8(2) |
| O1-Si1-C30-C32 | 71.4(3) |
| O1-C1-C6-C5 | 177.8(2) |
| O1-C1-C2-C3 | -178.2(2) |
| C5-O2-C7-C13 | 178.6(2) |
| C5-O2-C7-C8 | -0.6(3) |
| C5-C4-C3-C2 | 0.1(4) |
| C5-C4-C9-C8 | 0.4(4) |
| C7-O2-C5-C6 | -176.9(2) |
| C7-O2-C5-C4 | 2.5(3) |
| C7-C13-C14-C15 | -174.7(3) |
| C7-C13-C12-C11 | -15.8(4) |
| C7-C8-C9-C4 | 1.4(4) |
| C7-C8-C10-C11 | 27.7(5) |
| C1-C2-C3-C4 | 0.5(4) |
| C6-C5-C4-C3 | -0.6(4) |
| C6-C5-C4-C9 | 177.0(2) |
| C6-C1-C2-C3 | -0.6(4) |
| C13-C7-C8-C9 | 179.5(3) |
| C13-C7-C8-C10 | -2.6(4) |
| C13-C14-C15-C16 | -173.9(3) |
| C13-C14-C15-C17 | 5.5(5) |
| C13-C12-C11-C10 | 42.4(4) |
| C18-Si1-O1-C1 | -167.4(2) |
| C18-Si1-C24-C25 | -84.3(2) |
| C18-Si1-C24-C29 | 93.7(2) |
| C18-Si1-C30-C33 | -54.3(3) |
| C1-Si1-C30-C31 | 63.1(2) |
| C18-Si1-C30-C32 | -176.7(2) |
| C18-C23-C22-C21 | -0.9(5) |
| C18-C19-C20-C21 | -0.5(4) |
| C24-Si1-O1-C1 | 76.7(2) |
| C24-Si1-C18-C23 | -45.7(2) |
| C24-Si1-C18-C19 | 135.8(2) |
| C24-Si1-C30-C33 | 71.8(3) |
| C24-Si1-C30-C31 | -170.7(2) |
| C24-Si1-C30-C32 | -50.5(3) |
| C24-C25-C26-C27 | -0.6(5) |
| C24-C29-C28-C27 | 0.1(5) |
| C4-C5-C6-C1 | 0.6(4) |
| C14-C13-C12-C11 | 160.9(3) |
| C2-C1-C6-C5 | 0.0(4) |
| C3-C4-C9-C8 | 177.7(3) |
| C12-C13-C14-C15 | 8.5(4) |
| C8-C7-C13-C14 | -179.7(2) |
| C8-C7-C13-C12 | -2.8(4) |
| C8-C10-C11-C12 | -48.5(5) |
| C9-C4-C3-C2 | -177.4(3) |
| C9-C8-C10-C11 | -154.6(3) |
| C23-C18-C19-C20 | 0.0(4) |
| C19-C18-C23-C22 | 0.7(4) |
| C19-C20-C21-C22 | 0.3(5) |
| C25-C24-C29-C28 | -1.1(4) |
| C30-Si1-O1-C1 | -49.6(2) |
| C30-Si1-C18-C23 | 83.4(2) |
| C30-Si1-C18-C19 | -95.1(2) |
| C30-Si1-C24-C25 | 149.0(2) |
| C29-C24-C25-C26 | 1.4(4) |
| C20-C21-C22-C23 | 0.4(5) |
| C26-C27-C28-C29 | 0.7(5) |
| C28-C27-C26-C25 | -0.5(5) |
| C10-C8-C9-C4 | -176.2(3) |

Table S4. Comparison of single-function probes for detecting F^-^.

| Probe | emission | LOD | bio-imaging | References |
| --- | --- | --- | --- | --- |
|  | 485 nm | 1 μM | MCF-7/COS-7 cells | [2] |
|  | 740 nm | 0.2 μM | Hep G2 cells | [3] |
|  | 461 nm | 388 μM | A549 cells | [4] |
|  | 718 nm | 85 nM | none | [5] |
|  | 560 nm | 5.26 μM | none | [6] |
|  | 558 nm | 0.08 mM | RAW 264.7 cells | [7] |
|  | 450 nm | 1.0 μM | none | [8] |
|  | 526 nm | 1 mM | HeLa / L929 cells | [9] |
|  | 504 nm | 90 nM | HeLa cells | [10] |
|  | 410 nm | 1.0 μM | none | [11] |
|  | 650 nm | 14 nM | Huh7 cells and mice | This work |

Table S5. Comparison of single-function probes for detecting HClO.

| Probe | emission | LOD | bio-imaging | References |
| --- | --- | --- | --- | --- |
|  | 450 nm | 1.61 μM | none | [12] |
|  | 740 nm | 0.43 nM | RAW264.7 cells | [13] |
|  | 523 nm | 17.5 nM | HeLa cells | [14] |
|  | 416 nm | 35 nM | HeLa cells | [15] |
|  | 575 nm | 17.3 nM | HeLa  and J774A.1 cells | [16] |
|  | 427 nm | 0.21 μM | none | [16] |
|  | 506 nm | none | none | [17] |
|  | 510 nm | 56 nM | PC12 cells | [18] |
|  | 500- nm | 0.18 μM | none | [19] |
|  | 488 nm | 1.97 μM | none | [20] |
|  | 460 nm | 3.19 μM | Huh7 cells and mice | This work |

Table S6. Comparison of dual-function probes for detecting F^-^ and HClO.

| Probe | Two-channel | Peak spacing | Sequential response | NIR | bio-imaging | References |
| --- | --- | --- | --- | --- | --- | --- |
|  | Yes | 141 nm | Yes | No | HepG2 cells and mice | [21] |
|  | Yes | 365 nm | No | Yes | HepG2 cells and mice | [22] |
|  | Yes | 98 nm | Yes | No | HepG2 cells | [23] |
|  | Yes | 190 nm | Yes | Yes | Huh7 cells and mice | This work |

**References**

1.	Richard J-A: **De novo synthesis of phenolic dihydroxanthene near-infrared emitting fluorophores**. *Organic & Biomolecular Chemistry* 2015, **13,** 8169-8172.

2.	Zhang S, Fan J, Zhang S, Wang J, Wang X, Du J, Peng X: **Lighting up fluoride ions in cellular mitochondria using a highly selective and sensitive fluorescent probe**. *Chem Commun* 2014, **50,** 14021-14024.

3.	Tian X, Tong X, Li Z, Li D, Kong Q, Yang X: **In Vivo Fluoride Ion Detection and Imaging in Mice Using a Designed Near-Infrared Ratiometric Fluorescent Probe Based on IR-780**. *Journal of Agricultural and Food Chemistry* 2018, **66,** 11486-11491.

4.	Kim SY, Park J, Koh M, Park SB, Hong J-I: **Fluorescent probe for detection of fluoride in water and bioimaging in A549 human lung carcinoma cells**. *Chemical Communications* 2009, **31**, 4735-4737.

5.	Cao J, Zhao C, Zhu W: **A near-infrared fluorescence chemodosimeter for fluoride via specific Si–O cleavage**. *Tetrahedron Letters* 2012, **53,** 2107-2110.

6.	Hu R, Feng J, Hu D, Wang S, Li S, Li Y, Yang G: **A Rapid Aqueous Fluoride Ion Sensor with Dual Output Modes**. *Angewandte Chemie International Edition* 2010, **49,** 4915-4918.

7.	Zhu B, Yuan F, Li R, Li Y, Wei Q, Ma Z, Du B, Zhang X: **A highly selective colorimetric and ratiometric fluorescent chemodosimeter for imaging fluoride ions in living cells**. *Chemical Communications* 2011, **47,** 7098-7100.

8.	Bao Y, Liu B, Du F, Tian J, Wang H, Bai R: **A new strategy for highly selective fluorescent sensing of F^−^ and Zn^2+^ with dual output modes**. *Journal of Materials Chemistry* 2012, **22,** 5291-5294.

9.	Zheng F, Zeng F, Yu C, Hou X, Wu S: **A PEGylated Fluorescent Turn‐On Sensor for Detecting Fluoride Ions in Totally Aqueous Media and Its Imaging in Live Cells**. *Chemistry – A European Journal* 2012, **19,** 936-942.

10.	Jiang G, Liu X, Wu Y, Wang J, Dong X, Zhang G, Li Y, Fan X: **An AIE based tetraphenylethylene derivative for highly selective and light-up sensing of fluoride ions in aqueous solution and in living cells**. *RSC Advances* 2016, **6,** 59400-59404.

11.	Bao Y, Liu B, Wang H, Tian J, Bai R: **A “naked eye” and ratiometric fluorescent chemosensor for rapid detection of F− based on combination of desilylation reaction and excited-state proton transfer**. *Chemical Communications* 2011, **47,** 3957-3959.

12.	An N, Wang D, Zhao H, Gao Y: **A spectroscopic probe for hypochlorous acid detection**. *Spectrochimica Acta Part A: Molecular and Biomolecular Spectroscopy* 2022, **267,** 120529.

13.	Wu W-L, Zhao Z-M, Dai X, Su L, Zhao B-X: **A fast-response colorimetric and fluorescent probe for hypochlorite and its real applications in biological imaging**. *Sensors and Actuators B: Chemical* 2016, **232,** 390-395.

14.	Li J, Yang X, Zhang D, Liu Y, Tang J, Li Y, Zhao Y, Ye Y: **A fluorescein-based “turn-on” fluorescence probe for hypochlorous acid detection and its application in cell imaging**. *Sensors and Actuators B: Chemical* 2018, **265,** 84-90.

15.	Ma J, Jiao Z, Zhao M, Kong X, Xie H, Zhang Z: **A fluorescence probe for monitoring toxic hypochlorous acid in biosystems and environmental waters with a broad pH adaptation**. *Ecotoxicology and Environmental Safety* 2024, **288,** 117370.

16.	Feng H, Zhang Z, Meng Q, Jia H, Wang Y, Zhang R: **Rapid Response Fluorescence Probe Enabled In Vivo Diagnosis and Assessing Treatment Response of Hypochlorous Acid‐Mediated Rheumatoid Arthritis**. *Advanced Science* 2018, **5,** 1800397.

17.	Wang Y, Liu X, Li X: **Identifying the role of excited‐state proton transfer and photoinduced electron transfer in detecting hypochlorous acid for a benzothiazole‐based colorimetric fluorescent probe**. *Journal of Physical Organic Chemistry* 2022, **36,** e4461.

18.	Wang E, Qiao H, Zhou Y, Pang L, Yu F, Zhang J, Ma T: **A novel “turn-on” fluorogenic probe for sensing hypochlorous acid based on BODIPY**. *RSC Advances* 2015, **5,** 73040-73045.

19.	Sheng Y, Xu K, Wang Z, Gao Y: **A colorimetric and ratiometric fluorescent probe of hypochlorous acid and its bio-imaging application**. *Photochemical & Photobiological Sciences* 2024, **24,** 65-77.

20.	Yin H, Chi H, Shang Z, Qaitoon A, Yu J, Meng Q, Zhang Z, Jia H, Zhang R: **Development of a new water-soluble fluorescence probe for hypochlorous acid detection in drinking water**. *Food Chemistry: Molecular Sciences* 2021, 2, 100027.

21.	Feng T, Li D, Yang J, Yang T, Wu T, Zhu W, Wang Y, Hu T, Yan L, Le Y *et al*: **Development of a novel fluorescent probe for the Selective and sequential imaging of fluoride and hypochlorite**. *Spectrochimica Acta Part A: Molecular and Biomolecular Spectroscopy* 2025, **329,** 125578.

22.	Mei Y, Hai Z, Li Z, Rong K, Tang W, Song Q-H: **Dual-Responsive Near-Infrared BODIPY-Based Fluorescent Probe for the Detection of F– and HClO in Organisms**. *Analytical Chemistry* 2024, **96,** 3802-3809.

23.	Wei M, Long R, Liu R, Zheng L, Wang S, Jiang Y, Gu B, Niu L: **A ratiometric AIE probe with sequential activation for selective dual detection and imaging of fluoride and hypochlorous acid**. *Analytical and Bioanalytical Chemistry* 2025, **417,** 5487-5497.
